# Construction of high-density bin genetic map and QTL mapping of fruit aroma in longan (*Dimocarpus longan* Lour.)

**DOI:** 10.3389/fpls.2025.1642854

**Published:** 2025-08-14

**Authors:** Wenshun Hu, Qing Zhang, Chaojun Deng, Qizhi Xu, Jimou Jiang, Xiuping Chen, Jianguo Li, Jisen Zhang, Shaoquan Zheng

**Affiliations:** ^1^ Fruit Research Institute, Fujian Academy of Agricultural Sciences, Fujian Breeding Engineering Technology Research Center for Longan and Loquat, Fuzhou, China; ^2^ State Key Lab for Conservation and Utilization of Subtropical Agro-Biological Resources, College of Agriculture, Guangxi University, Nanning, China; ^3^ College of Horticulture, South China Agricultural University, Guangzhou, China

**Keywords:** longan, high-density bin map, characteristic aroma components, QTL mapping, candidate gene

## Abstract

Aroma is a crucial factor influencing the flavor quality and economic value of longan fruits. This study employed a mapping population consisting of 98 F_1_ progeny of ‘Shixia × Xiangcui’ (exhibiting broad segregation in fruit aroma trait) and their parents. We performed the SNP genotyping through whole-genome resequencing to construct a high-density linkage map, followed by QTL mapping and candidate gene screening for aroma trait. We obtained a total of 554.9 Gbp of sequencing data, with an average depth of approximately 15× for the parents and 12× for the progeny. Three types of SNP markers (lm×ll, nn×np, and hk×hk) were developed, totaling 317 877. After merging with a 100 kb sliding window, 6 134 Bin markers were generated. A first high-density Bin map was constructed, comprising 15 linkage groups with 3 517 Bin markers (containing 264 385 SNPs), covering a total map length of 1 666.79 cM. The average marker interval was 0.48 cM, with 99.18% of gaps being less than 5 cM. Collinearity analysis confirmed the high quality of the map. Fifty-six QTLs were mapped for 9 aroma-related traits, including (E)-2-hexenal, ethyl acetate, ethyl butyrate, ethyl crotonate, (E)-2-hexenoate, ethanol, linalool, ocimene, and total ester content. These QTLs explained 19.8%–51.0% of the phenotypic variation rates and were distributed across 11 linkage groups, with two QTL-rich regions on LG7 and LG8. Seven pleiotropic QTLs were detected. By analyzing the expression patterns of 1 535 annotated genes within the mapped intervals, six candidate genes potentially regulating the synthesis of ester characteristic aroma compounds were identified: aldo-keto reductase AKRs, acetolactate synthase small subunit ALS2, ACC oxidase ACO1-1, and transcription factors bHLH122, AGL103, and bZip1. This work provides novel insights into the fruit aroma formation, and facilitates breeding efforts to improve quality in longan and potentially other fruit crops.

## 1 Introduction

Longan (*Dimocarpus longan* Lour.) is a renowned specialty fruit in tropical and subtropical regions of China and Southeast Asia, occupies approximately ~5.7 million hectares globally with annual production reaching ~4.2 million tons. China is the origin and the leading producer of longan (Zheng et al., 2019), but cultivation is dominated by two varieties, ‘Shixia’ and ‘Chuliang’, which account for about 80% of the national acreage, and the fruit also lacks fragrance. In contrast, the ‘Chompoo’, ‘Biew Kiew’, and ‘E-Daw’ cultivated in Thailand, as well as the ‘Kohala’ grown commercially in the United States and Australia, are all well-known strong fragrant longan varieties in the international fruit market. Therefore, deepening the understanding of the molecular mechanisms underlying longan fruit aroma formation and related breeding research has important significance.

Genetic mapping, also known as linkage mapping, is an effective method for locating genes associated with important economic traits. The construction of genetic maps and QTL mapping for economic traits in longan (2n=2x=30) started relatively late and has evolved through two stages: reference-free and reference-based mapping. Guo et al. (Guo et al., 2009) used 94 F_1_ progenies of ‘Fengliduo × Dawuyuan’ as mapping population, and constructed the first molecular genetic map of longan by RAPD, ISSR, SRAP, and AFLP markers. The maternal and paternal maps contained 21 and 22 linkage groups, with 183 and 251 marker loci, respectively, and average intervals of 5.84 cM and 4.65 cM. Subsequent studies based on this map identified QTLs for traits such as single fruit weight, soluble solid content, seed weight, peel weight, and edible rate (Guo et al., 2011), as well as trunk circumference (Guo et al., 2012). After the release of the first longan reference genome (at the contig level) (Lin et al., 2017), high-throughput SNP detection methods were applied to linkage mapping. Jue et al. (Jue et al., 2021) expanded the ‘Fengliduo × Dawuyuan’ population to 200 F_1_ progeny and used RAD-Seq to construct a high-density SNP genetic map with 15 linkage groups (first consistent with chromosome count), 8014 SNPs, and an average interval of 0.36 cM. This map enabled the identification of 17 stable QTLs for single fruit weight and edible rate across years, along with 3 candidate genes. However, the above studies have limitations such as the use of a single hybrid combination, low density of traditional markers like RAPD, or the absence of a chromosome-level reference genome, which hinder the accurate localization of the map.

Currently, high-throughput sequencing can easily generate millions of SNP markers, but this volume exceeds the computational capacity of commonly used mapping software. The recombination breakpoint strategy addresses this by fusing identical genotypes within the sliding window into Bin markers, reducing marker redundancy while retaining complete genetic information. Compared to RFLP, SSR, InDel, or single SNP markers, Bin markers offer greater informativeness for a given population, and the sliding window approach helps eliminate false positives caused by sequencing errors. In recent years, high-density Bin genetic map construction and QTL mapping have been applied to various annual crops such as rice (Zhao et al., 2022), soybean (Ma et al., 2023), maize (Zhou et al., 2016), rapa (Li F. M. et al., 2023), and melon (Pereira et al., 2018), as well as a few woody fruit crops like apple (Di Pierro et al., 2016) and pear (Qin et al., 2022).

Aroma is a typical quantitative trait controlled by multiple genes. High-density genetic maps have proven effective for QTL mapping of aroma trait in annual crops like rice (Amarawathi et al., 2008), wheat (Kiszonas et al., 2017), tomato (Tikunov et al., 2020), cucumber (Sun et al., 2022), and strawberry (Rey-Serra et al., 2022). Perennial fruit trees face challenges such as long breeding cycles, large space requirements, and the complexity of aroma compound identification and quantification. However, with the increasing attention to fruit quality, progress has been made in aroma-related QTL mapping for kiwifruit (Zeng et al., 2020; Souleyre et al., 2022), grape (Koyama et al., 2022), apple (Yang et al., 2023), peach (Eduardo et al., 2013; Sánchez et al., 2014), and citrus (Yu et al., 2017). To date, no studies have reported Bin map construction and aroma-related QTL mapping in longan. Based on the chromosome-level reference genome of the ‘Shixia’ cultivar, this study constructed a high-density Bin-marker linkage map through whole-genome resequencing of an F_1_ population derived from a ‘Shixia × Xiangcui’ cross. This map was then leveraged to identify aroma-related QTLs/candidate genes, ultimately aiming to elucidate the molecular mechanisms of aroma formation in longan fruit and support marker-assisted breeding.

## 2 Materials and methods

### 2.1 Plant material

**Figure 1:**
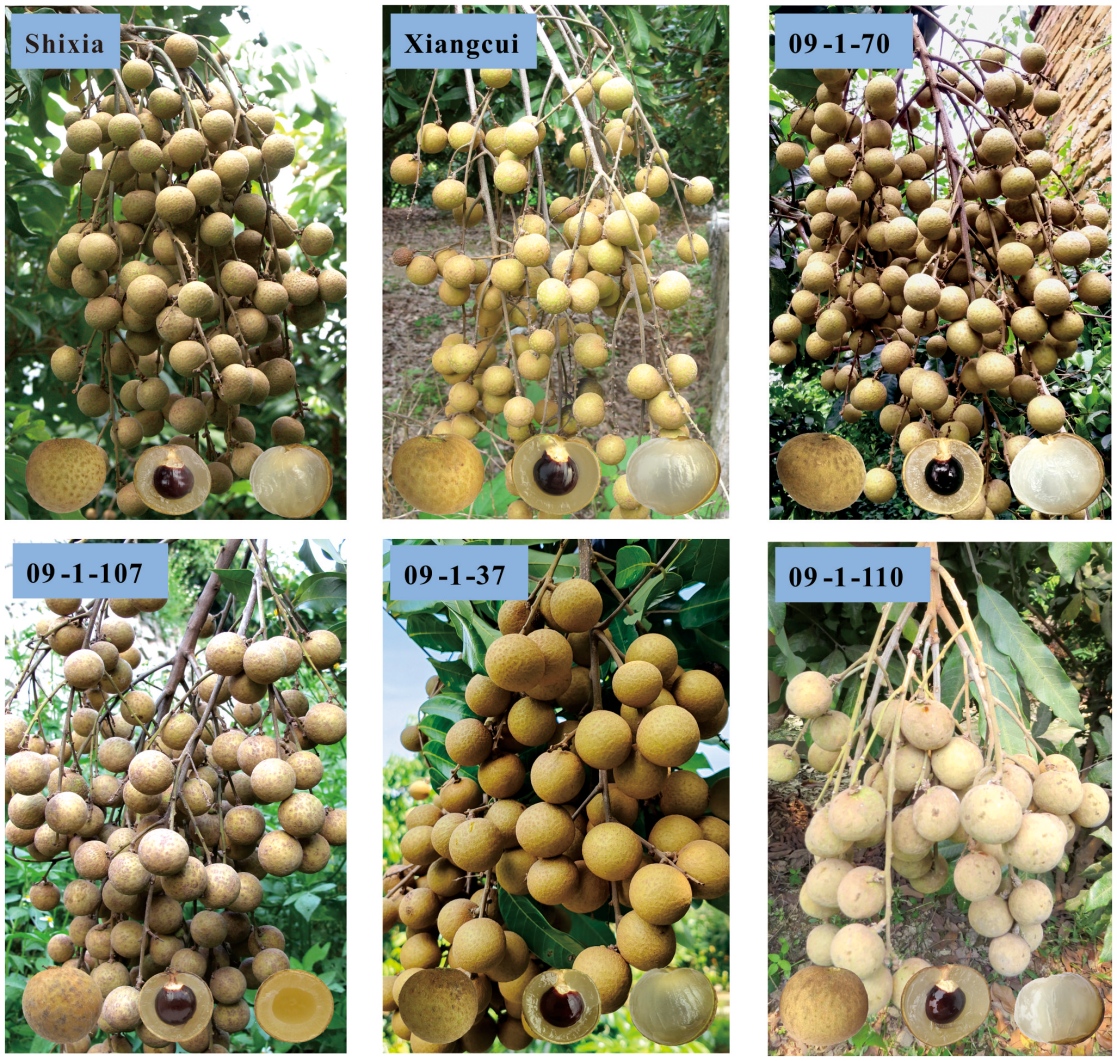
Ear trait of parental lines and partial F_1_ hybrids progeny. Progeny with light fruit aroma: 09-1-70, and 09-1-107; Progeny with strong fruit aroma: 09-1-37; Progeny without fruit aroma: 09-1-110.

With high yield, premium quality and rich aroma as the breeding objectives, in 2009, the non-aromatic early-maturing cultivar ‘Shixia’ (a major domestic cultivar) and the aromatic late-maturing large-fruit cultivar ‘Xiangcui’ (a bud sport of Thailand’s major cultivar ‘Biew Kiew’) were cross-pollinated to obtain the F_1_ progeny. The fruit aroma of the offsping exhibits a wide variation, including none, weak and strong (Hu et al., 2015). For this study, the parental lines and 98 F_1_ offspring grafted on trees were selected as the mapping population (**Figure 1**).

### 2.2 Experimental methods

#### 2.2.1 Extraction and detection of genome DNA

Young leaves were collected, and genomic DNA was extracted by modified CTAB method. DNA integrity was assessed via 1% agarose gel electrophoresis, and the concentration was measured using a Nanodrop spectrophotometer. Qualified DNA samples were used for high-throughput sequencing.

#### 2.2.2 Resequencing library construction and sequencing

The NEBNext Ultra DNA Library Prep Kit was used to construct whole-genome resequencing libraries of 100 samples from the parents ‘Shixia’, ‘Xiangcui’ and their 98 F_1_ offspring. The Qualified libraries were sequenced on the Illumina HiSeq 2500 platform at the Fujian Agriculture and Forestry University Genomics and Biotechnology Research Center. The sequencing modes for the hybrid progeny and parents were High-throughput 1×100 nt and High-throughput 2×100 nt, respectively, with a sequencing depth of >10×.

#### 2.2.3 SNP detection

Raw reads from the 100 resequenced samples were quality-controlled using Trimmomatic (Bolger et al., 2014) to remove low-quality reads and adapter sequences. Clean reads were aligned to the ‘Shixia’ reference genome (https://www.ncbi.nlm.nih.gov/bioproject/PRJNA741049/) using BWA (Jo and Koh, 2015) with default parameters. PCR duplicates were marked and removed using Picard (http://sourceforge.net/projects/picard/). SNP calling and filtering were performed using GATK (https://github.com/broadinstitute/gatk/releases) and VCFtools (Danecek et al., 2011) to obtain a SNP dataset.

#### 2.2.4 SNP genotyping and genetic map construction

The SNP dataset was filtered to remove markers located on non-chromosomal contigs. Markers were classified into three types based on parental genotypes: lm×ll, nn×np, and hk×hk. Chi-square tests (*P* < 0.05) were applied to retain markers with segregation ratios of 1:1, 1:1, and 1:2:1, respectively.

To reduce redundancy while retaining sufficient genetic information, Bin markers were created using the Slide window method in Binmarker-v2.3 (). Methods such as recombination breakpoints determination, genotyping error correction, and window merging of the same genotype were referred to Qin et al. (Qin et al., 2022).

According to the principle of ‘double false testcross’, the CP (Cross Pollinator) model in JoinMap 4.1 was used for genetic map construction through a systematic workflow. First, the three types of Bin markers were combined and loaded into the software, and the population was created after checking the data format. Second, parameters included regression mapping, Kosambi mapping function, a single iteration, and a minimum LOD score of 10.0 for linkage group clustering. Third, markers with severe segregation distortion (χ² test, *P* < 0.05), missing rates ≥10%, and similarity = 1.0 were removed. Fourth, nodes of linkage group were determined, and the map was drawn after three iterations. MapChart 2.2 was used for visualization (Voorrips, 2002), and ALLMAPs assessed the consistency between the Hi-C-based chromosome-level genome and the genetic map (Tang et al., 2015).

#### 2.2.5 Data acquisition of fruit aroma trait in hybrid population

Prior research by our group identified 22 characteristic aroma compounds through volatile profiling of parental fruits across developmental stages. Comprehensive phenotypic datasets were obtained, including: five-year aroma sensory evaluations (2012–2015 and 2018, 83 progeny) and volatile compound GC-MS detection (2018, 48 progeny), which will be published separately. For the present QTL mapping study, we selected ten key traits: (E)-2-hexenal (A6), ethyl acetate (E1), ethyl butyrate (E3), ethyl crotonate (E4), ethyl (E)-2-hexenoate (E7), ethanol (AO1), ocimene (M1), linalool (M5), total ester content (TE), and 5-year comprehensive value of aroma sensory evaluation (FS). The content of E1, E3, E4, E7, AO1, and TE were significantly correlated with aroma intensity, and A6, M1, and M5 were the main characteristic aroma components.

#### 2.2.6 QTL mapping for aroma traits

QTL analysis was performed using MapQTL 6.0 (Van Ooijen, 2009). Normally distributed traits were analyzed using Interval Mapping, while others used the Kruskal-Wallis (KW) test (Qin et al., 2022). Missing phenotypic data for individual plants were treated as null values. Significance thresholds were established through 10 000 permutation tests (PT), with LOD scores determined as follows: primary threshold calculation considered genome-wide confidence levels (0.95 or 0.90), adopting the more stringent value; if no significant interval was detected, linkage group-specific thresholds (0.95 or 0.90 confidence) were applied, again selecting the higher value. The minimum initial LOD threshold was set at ≥3.0. Bin markers meeting these criteria were designated as putative QTLs. The ‘1 LOD-drop’ method (Lander and Botstein, 1989) defined QTL confidence intervals, and phenotypic variance explained (PVE) was calculated for each QTL. QTLs were named as ‘trait code + peak Bin marker’ (e.g., M1-hk0303 denotes an ocimene-related QTL with peak signal at marker hk0303).

#### 2.2.7 Candidate gene identification

QTL intervals from the genetic map were anchored to physical genomic of ‘Shixia’ genome for gene retrieval and functional annotation. The expression level of gene was derived from prior RNA-seq data (a total of 17 developmental stages of ‘Shixia’, ‘Xiangcui’, and ‘Lidongben’ cultivars), and the mean FPKM < 0.5 genes were filtered out. Subsequent KEGG enrichment analysis was performed using ClusterProfiler in R package (Yu et al., 2012). The goodSamplesGenes function of the WGCNA package was used to detect and remove genes with low-variance (standard deviation ≤ 0.5). Gene correlations were calculated via biweight mid-correlation, with soft-thresholding power (β) determined by pickSoftThreshold (Peter and Steve, 2008). Modules were detected (minModuleSize = 20) and merged (mergeCutHeight = 0.25), followed by co-expression network visualization in Cytoscape v3.7.1 (Shannon et al., 2003). Pearson correlations between gene expression and characteristic aroma compounds were analyzed using SPSS 25. Key candidate genes were screened by referring to metabolic pathway, gene function, and literature report etc.

## 3 Results

### 3.1 Resequencing of the hybrid population and SNP detection

Whole-genome resequencing of the two longan parents and 98 F_1_ progeny generated a total of 554.9 Gb of raw data (**Table 1**). The parental cultivars ‘Shixia’ and ‘Xiangcui’ yielded 7.1 Gb and 6.9 Gb of data, respectively, and the sequencing amount of the F_1_ population was 3.9-7.1 Gb. The Q30 values of all samples exceeded 90%, indicating high base accuracy. After filtering, the clean reads were aligned to the ‘Shixia’ reference genome (about 483 Mb). The sequencing depth reached 15.47× for the maternal parent ‘Shixia’ and 15.03× for the paternal parent ‘Xiangcui’, and the average sequencing depth of hybrid offspring was 12.25×.

**Table 1:** The sequences data of parents and progenies.

Sample	Total reads	Total bases (Gbp)	Q30 percentage (%)	Ave-depth (×)
Shixia	70 013 962	7.1	92.81	15.47
Xiangcui	67 986 648	6.9	94.11	15.03
Progenies	37 268 146-70 436 894	3.9-7.1	91.76-95.10	12.25
Total	5 493 228 903	554.9	/	/

Q30 Percentage refers to the percentage of bases with sequencing quality values greater than or equal to 30.

**Figure 2:**
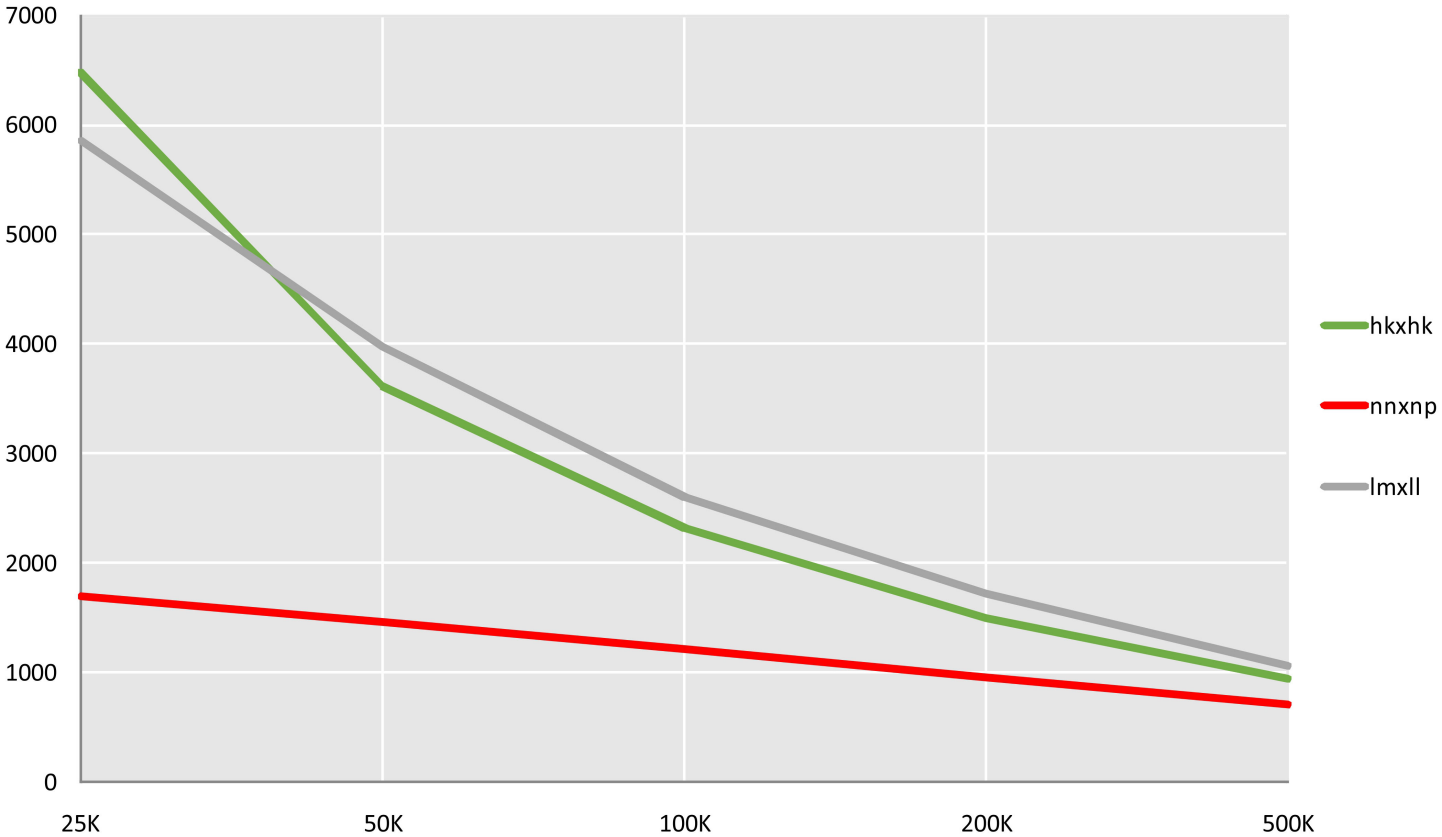
Quantity statistics of Bin markers in slide windows of different length. The X-axis represents the length of the slide window, and the Y-axis represents the number of markers.

Through GATK SNP calling and VCFtools filtering, 4 261 436 high-confidence SNP loci were obtained from 11 521 588 raw SNPs. Genotyping and subsequent screening (excluding markers with chi-square test *p*-values < 0.05 and missing rates ≥ 10%) yielded a total of 317 877 markers suitable for CP model mapping These comprised three types: 59,744 lm×ll (maternal heterozygous, paternal homozygous), 5,704 nn×np (maternal homozygous, paternal heterozygous), and 252,429 hk×hk (both parents heterozygous). The total number of these SNPs far exceeds the computational limits of general mapping software. Five sliding windows with different scales of 25, 50, 100, 200 and 500 kb were used to compare the fusion of three types of markers. and the 100 kb sliding window, which produced 6 134 Bin markers, was determined to be optimal (**Figure 2**). These Bin markers ranged in length from 1 bp to 874 474 bp (average 58.4 kb) and contained 1 to 1 791 internal SNPs (average 52 SNPs). Joinmap was then used to re-eliminate markers with severe segregation distortion (chi-square test *P* < 0.01) and missing rate ≥ 10% for three types of markers, and 5 626 Bin markers were obtained for final mapping, of which lm × ll, nn × np, hk × hk were 2 414, 1 189, and 2 023, respectively.

### 3.2 Construction and evaluation of the high-density genetic map

**Figure 3:**
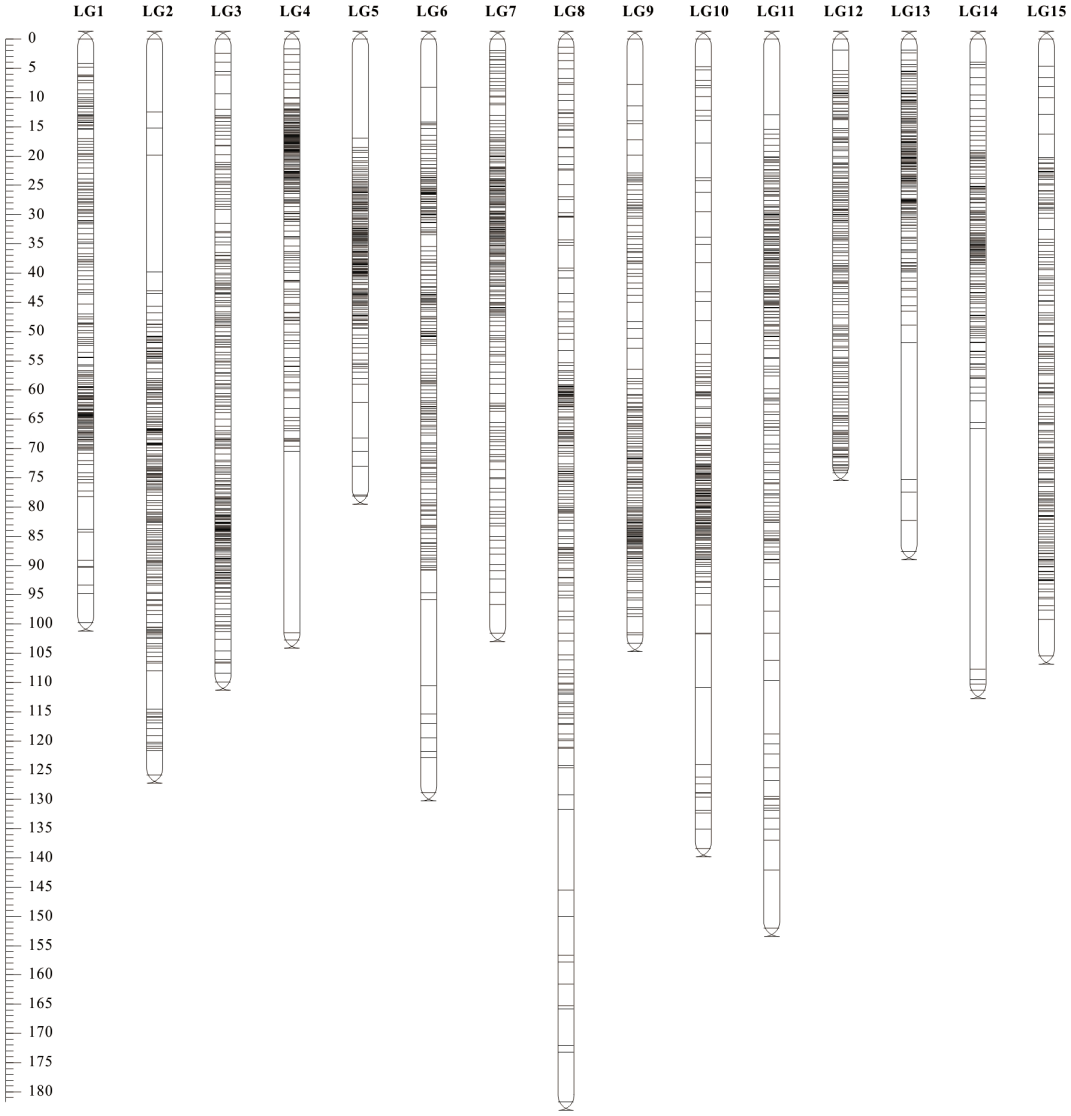
High-density SNP genetic map of longan.

The three types of final markers obtained above were loaded together into Joinmap 4.1 software for grouping, and 15 strong linkage groups (LGs) were selected for map construction. A total of 3 517 Bin markers were ultimately mapped, comprising 1 407 lm×ll, 394 nn×np, and 1 716 hk×hk markers, which collectively contained 264,385 SNPs (**Figure 3**; **Table 2**; **Supplementary Figure S1**). The lengths of the 15 linkage groups ranged from 74.12 cM (LG12) to 181.74 cM (LG8). The number of Bin markers per LG varied from 193 (LG15) to 276 (LG3), and the number of SNP markers ranged from 7 666 (LG5) to 29 368 (LG12). The proportion of gaps <5 cM per linkage group ranged from 98.09% (LG10) to 100% (LG3, LG7, LG12). Overall, the map spanned a total length of 1 666.79 cM, with an average marker interval of 0.48 cM. The proportions of gaps <5 cM and <1 cM reached 99.18% and 90.89%, respectively, indicating uniform marker distribution and small intervals.

**Table 2:** SNP genetic map information of longan constructed based on resequencing.

Link group	Length (cM)	Anchored Bin	Anchored SNP	Average distance (cM)	ρ value	Max Gap (cM)	Gap<5 cM (%)
LG1	99.82	251	19 341	0.40	0.921	5.65	99.20
LG2	125.76	224	21 850	0.56	0.865	19.92	98.66
LG3	109.92	276	18 509	0.40	0.904	3.25	100.00
LG4	102.68	268	20 942	0.38	0.820	30.99	99.63
LG5	78.09	238	7 666	0.33	0.578	16.88	99.16
LG6	128.77	248	18 593	0.52	0.923	14.77	98.39
LG7	101.65	247	10 567	0.41	0.869	4.98	100.00
LG8	181.74	233	19 622	0.78	0.861	13.72	98.28
LG9	103.33	231	17 269	0.45	0.915	7.71	99.57
LG10	138.39	209	11 728	0.66	0.932	13.15	98.09
LG11	152.02	240	15 725	0.63	0.921	12.88	98.33
LG12	74.12	219	29 368	0.34	0.994	3.54	100.00
LG13	87.56	235	17 242	0.37	0.862	23.37	99.15
LG14	111.25	205	20 307	0.54	0.807	41.07	99.51
LG15	105.54	193	15 656	0.55	0.879	6.31	99.48
Total	1 666.79	3 517	264 385	0.48	0.870	41.07	99.18

Visualization and evaluation using ALLMAPs software showed that the Spearman’s correlation coefficient (ρ) between the marker order on the genetic map and their corresponding positions on the physical map ranged from 0.578 to 0.994, with an average value of 0.870. Except for LG5, all linkage groups had ρ value greater than 0.8. Seven linkage groups (LG1, LG3, LG6, LG9, LG10, LG11, LG12) exhibited high collinearity with the genomic map in terms of marker order (**Table 2**; **Supplementary Figures S2**, **S3**), all exceeding ρ > 0.9. These results indicate that this linkage map is of high quality and suitable for subsequent QTL analysis.

### 3.3 Phenotypic data analysis of aroma traits

Genetic variation in progeny of nine aroma-related traits including (E)-2-hexenal (A6), ethyl acetate (E1), ethyl butyrate (E3), ethyl crotonate (E4), ethyl (E)-2-hexenoate (E7), ethanol (AO1), ocimene (M1), linalool (M5), and total ester content (TE) was analyzed. Results showed that all nine traits exhibited quantitative genetic characteristics with continuous and extensive variation (**Table 3**). Due to the large difference in content between different volatiles and the moderately positive skewness distribution (Z-score > 3), the log2 value was converted and then subjected to normality testing (**Table 3**). E1, E7, AO1, M1, and TE conformed to normal distributions.

**Table 3:** Phenotypic statistics of fruit aroma related traits.

Compounds	Parents			F_1_ population					
	Shixia	Xiangcui	Mid-parent value	Average	Range	CV (%)	Kurtosis	Skewness	*P*-value
A6	1248.4 ± 191.9	1155.8 ± 281.1	1202.1	413.6	31.8 - 2800.0	134.09	4.47	-1.81	0.003
E1	927.7 ± 284.8	4360.0 ± 1017.3	2643.8	229.0	26.3 - 792.0	73.61	-0.52	-0.21	0.639
E3	65.3 ± 15.2	345.2 ± 91.9	205.2	65.6	2.5 - 238.5	104.00	-0.71	-0.85	0
E4	252.5 ± 55.8	527.0 ± 108.7	389.7	132.9	11.5 - 841.5	120.56	1.26	-1.22	0
E7	6.8 ± 1.8	71.3 ± 33.5	39.1	11.4	1.6 - 70.1	117.47	0.49	-0.42	0.320
AO1	958.9 ± 210.0	1345.8 ± 271.4	1152.4	642.3	285.6 - 2030.7	43.14	1.81	0.56	0.148
M1	28750.4 ± 5375.8	19526.5 ± 5482.1	24138.4	13376.7	486.3 - 55309.7	97.66	-0.52	-0.36	0.310
M5	12.1 ± 11.9	38.2 ± 14.4	25.1	22.1	2.6 - 56.1	65.98	1.47	-1.06	0.004
TE	1629.0	7539.3	4584.2	778.9	225.6 - 1712.8	44.77	0.28	-0.50	0.134

The unit of volatile compound content was ng·g^-1^ FW. The Shapiro-Wilk method of SPSS 25 was used, a *P*-value > 0.05 indicates a normal distribution.

### 3.4 QTL mapping of characteristic aroma compounds

**Figure 4:**
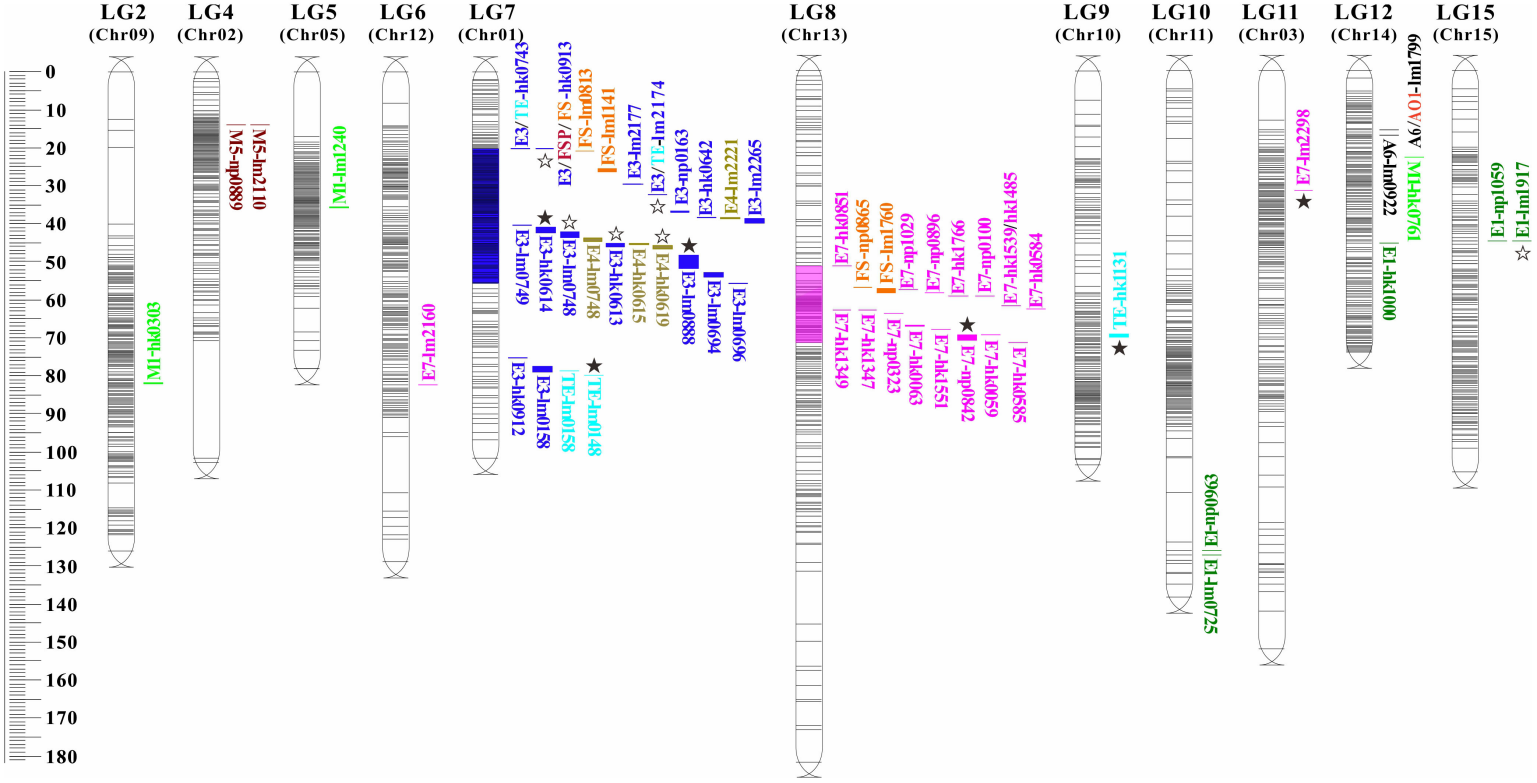
Distribution of QTLs related to characteristic aroma compounds in longan. The QTL loci for traits are named according to Table 3-3. Different colored markers represent different aroma compounds, and the blue and purple red segments labeled on linkage groups (LG) are the loci enrichment regions. The ☆ and ★ represent identified preliminary candidate genes and key candidate genes, respectively.

Using the high-density Bin map, 56 QTLs associated with nine aroma traits were identified. These QTLs were unevenly distributed across 11 linkage groups such as LG2, LG4, LG5, with significant enrichment observed on LG7 and LG8 (**Table 4**; **Figure 4**).

**Table 4:** QTL mapping results of key aroma compounds on the ‘Shixia×Xiangcui’ linkage map.

Trait	QTL	Confidence interval (cM)	Physical location (Mb)	LOD	Expl.%
A6	A6-lm1799	LG12: 15.639	Chr14: 2.159-2.248	5.02	38.9
	A6-lm0922	LG12: 16.935	Chr14: 5.554-5.644	5.64	42.4
AO1	AO1-lm1799	LG12: 15.639	Chr14: 2.159-2.248	3.70	30.4
E1	E1-np0963	LG10: 126.233	Chr11: 12.066-12.094	3.43	28.5
	E1-lm0725	LG10: 127.271	Chr11: 4.101-4.174	3.59	29.7
	E1-hk1000	LG12: 45.355	Chr14: 10.587-10.686	3.84	31.3
	E1-np1059	LG15: 44.663	Chr15: 5.002-5.202	3.73	30.6
	E1-lm1917	LG15: 44.743	Chr15: 8.713-8.789	3.72	30.5
E3	E3-hk0743	LG7: 19.961	Chr01: 27.407-27.610	4.19	33.7
	E3-hk0913	LG7: 20.136	Chr01: 28.772-29.772	4.52	35.8
	E3-lm2177	LG7: 29.404	Chr01: 21.179-21.266	4.45	35.4
	E3-lm2174	LG7: 32.137-32.179	Chr01: 19.981-21.849	3.91	31.8
	E3-np0163	LG7: 36.570-36.813	Chr01: 26.639-28.697	4.73	37.1
	E3-hk0642	LG7: 38.064-38.202	Chr01: 28.502-30.569	4.98	38.6
	E3-lm2265	LG7: 38.556-39.562	Chr01: 29.725-31.043	7.29	51.0
	E3-lm0749	LG7: 40.112	Chr01: 32.249-32.348	4.26	34.1
	E3-hk0614	LG7: 40.724-42.156	Chr01: 30.413-31.393	4.97	38.6
	E3-lm0748	LG7: 42.287-43.929	Chr01: 32.127-33.633	5.54	41.9
	E3-hk0613	LG7: 44.951-46.037	Chr01: 33.416-34.025	4.57	36.1
	E3-lm0888	LG7: 48.225-51.648	Chr01: 35.161-35.890	5.43	41.2
	E3-lm0694	LG7: 52.749-53.890	Chr01: 36.262-36.512	4.57	36.1
	E3-lm0696	LG7: 55.629	Chr01: 35.986-36.031	4.30	34.4
	E3-hk0912	LG7: 75.238	Chr01: 29.552-29.613	5.02	38.8
	E3-lm0158	LG7: 77.512-78.803	Chr01: 40.484-42.168	4.41	35.1
E4	E4-lm2221	LG7: 38.172-38.365	Chr01: 29.827-30.569	3.81	31.2
	E4-lm0748	LG7: 43.690-44.383	Chr01: 33.360-33.734	3.87	31.5
	E4-hk0615	LG7: 44.951-45.336	Chr01: 33.529-34.025	3.85	31.5
	E4-hk0619	LG7: 45.473-46.427	Chr01: 33.416-34.135	3.75	30.8
E7	E7-lm2160	LG6: 82.093	Chr12: 2.190-2.207	4.26	34.1
	E7-hk0851	LG8: 51.262	Chr13: 21.015-21.105	4.15	33.4
	E7-np1029	LG8: 57.475	Chr13: 18.748-18.766	5.87	43.7
	E7-np0896	LG8: 58.440	Chr13: 25.310-25.376	5.08	39.2
	E7-hk1766	LG8: 59.093	Chr13: 19.150-19.153	4.09	33.0
	E7-np0100	LG8: 59.298	Chr13: 17.213-17.307	4.32	34.5
	E7-hk1539	LG8: 61.840	Chr13: 22.236-22.328	4.48	35.5
	E7-hk1485	LG8: 61.840	Chr13: 24.263-25.207	4.48	35.5
	E7-hk0584	LG8: 62.610	Chr13: 13.993-14.193	4.53	35.8
	E7-hk1349	LG8: 62.779	Chr13: 16.487-16.584	4.53	35.8
	E7-hk1347	LG8: 62.779-62.947	Chr13: 15.219-15.538	4.53	35.8
	E7-np0323	LG8: 63.694	Chr13: 13.791-13.791	4.82	37.6
	E7-hk0063	LG8: 66.881-67.309	Chr13: 13.853-14.097	5.19	39.9
	E7-hk1551	LG8: 67.901-68.131	Chr13: 13.435-13.651	4.57	36.1
	E7-hk0059	LG8: 69.492	Chr13: 14.198-14.298	4.82	37.7
	E7-np0842	LG8: 69.442-70.795	Chr13: 11.707-12.459	5.52	41.8
	E7-hk0585	LG8: 71.286	Chr13: 16.596-16.690	4.54	35.9
	E7-lm2298	LG11: 31.329	Chr03: 38.249-39.182	4.17	33.6
M1	M1-hk0303	LG2: 81.568-81.957	Chr09: 10.737-11.04	3.53	29.2
	M1-lm1240	LG5: 35.553	Chr05: 17.836-18.038	3.25	27.3
	M1-hk0761	LG12: 23.789	Chr14: 4.922-5.020	3.26	27.4
M5	M5-np0889	LG4: 13.690	Chr02: 8.563-8.625	3.21	27.0
	M5-lm2110	LG4: 13.743	Chr02: 4.025-4.084	3.52	29.1
TE	TE- hk0743	LG7: 19.961	Chr01: 27.407-27.610	4.01	32.5
	TE-lm2174	LG7: 32.137-32.179	Chr01: 19.981-21.849	3.57	29.5
	TE-lm0158	LG7: 78.803	Chr01: 42.036-42.268	3.65	30.0
	TE-lm0148	LG7: 80.028	Chr01: 44.890-45.172	3.80	31.1
	TE-hk1131	LG9: 68.832-69.602	Chr10: 14.920-17.224	4.75	37.2
FSP	FSP-lm0687	LG1: 54.359-54.518	Chr07: 17.568-18.568	3.45	28.7
	FSP-hk0913	LG7: 20.136	Chr01: 28.772-29.772	6.56	47.4
FS	FS-lm0687	LG1: 54.359-54.518	Chr07: 17.568-18.568	3.95	21.8
	FS-hk0913	LG7: 20.136	Chr01: 28.772-29.772	3.58	20.0
	FS-lm0813	LG7: 20.947	Chr01: 12.301-12.401	3.80	21.1
	FS-lm1141	LG7: 25.539-26.398	Chr01: 14.619-15.236	3.82	21.2
	FS-np0865	LG8: 56.619	Chr07: 4.386-4.586	3.55	19.8
	FS-lm1760	LG8: 56.861-58.131	Chr07: 6.080-6.353	3.67	20.4

Expl.%-The percentage of the variance explained for by the QTL. TE-Total content of ester substances, FSP-Comprehensive value of five-year aroma sensory evaluation for partial plants (48 plants tested in 2018), FS- Comprehensive value of five-year aroma sensory evaluation (83 offspring plants).

Two QTLs for (E)-2-hexenal (A6) content were mapped on LG12, both exhibiting LOD scores >5.0 and explaining 38.9%-42.4% of phenotypic variation. One QTLs for ethanol (AO1) content was also detected on LG12 (30.4% PVE). Five QTLs for ethyl acetate (E1) content were localized to LG10, LG12, and LG15 (28.5%-31.3% PVE).

Sixteen QTLs for ethyl butyrate (E3) and four for ethyl crotonate (E4) were all mapped on LG7, and most of them were clustered within the LG7:19.961-55.629 cM region (blue zone, LOD=3.75-7.29, 30.8%-51.0% PVE). Eighteen QTLs for ethyl (E)-2-hexenoate (E7) were mapped on LG6, LG8, and LG11 (33.0%-43.7% PVE), with 16 concentrated in the LG8:51.262-71.286 cM segment (magenta zone). Three QTLs for ocimene (M1) on LG2, LG5, and LG12 explained 27.3%-29.2% PVE. Two adjacent QTLs for linalool (M5) on LG4 explained 27.0%-29.1% PVE. For total ester content (TE), four QTLs on LG7 and one on LG9, explaining 29.5%-37.2% PVE.

Additionally, six of eight QTLs for the 5-year comprehensive value of aroma sensory evaluation were located in the enrichment regions on LG7 and LG8 (**Table 4**). In summary, seven pleiotropic QTLs were identified across traits, including A6/AO1-lm1799 on LG12, FSP/FS-lm0687 on LG1, and E3/TE-hk0743, E3/FSP/FS-hk0913, E3/TE-lm2174, E3/E4-lm0748, and E3/TE-lm0158 on LG7.

### 3.5 Pathway enrichment and WGCNA analysis

**Figure 5:**
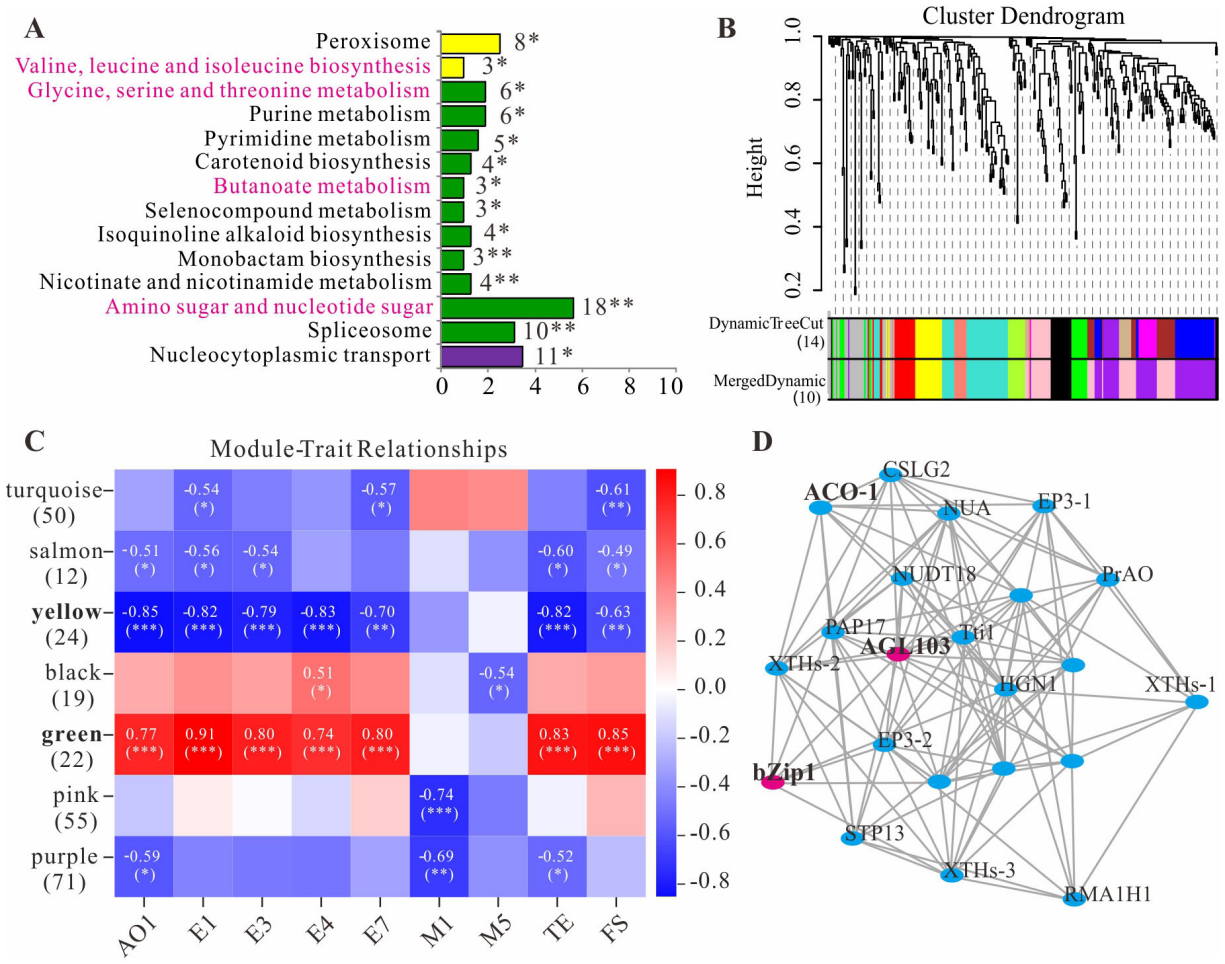
Pathway enrichment and WGCNA analysis of QTL mapping genes. **(A)**, KEGG enrichment pathways. **(B)**, Clustering dendrogram of genes and module division. **(C)**, Heat map of the relationship between modules and aroma traits. **(D)**, Gene co-expression networks within green module. The symbols *, ** and *** indicate statistical significance at *P* < 0.05, *P* < 0.01 and *P* < 0.001, respectively.

Within the physical map intervals corresponding to the 56 QTL loci (after deduplication), a total of 1 535 genes were retrieved, with 747 genes retained based on an FPKM value ≥ 0.5. KEGG analysis revealed that 319 genes were assigned to 134 pathways based on their functions (**Supplementary Table S3**). Among the 14 significantly enriched pathways, four pathways—Butanoate metabolism, Carotenoid biosynthesis, Amino sugar and nucleotide sugar metabolism, Valine, leucine and isoleucine biosynthesis, and Glycine, serine and threonine metabolism—were potentially related to volatile metabolite synthesis in longan fruit (**Figure 5A**). After filtering the 747 genes for variance in expression levels, WGCNA was performed on the 319 genes. When β=22, the average connectivity approached 0, so this β value was selected to construct the scale-free network (**Supplementary Figure S4**). The TOM algorithm was used to generate a gene hierarchical clustering tree. Gene modules were partitioned using the dynamic tree cut algorithm, and similar modules were subsequently merged, resulting in 10 final co-expression modules (**Figure 5B**). The gene count within modules ranged from a maximum of 71 in the purple module to a minimum of 12 in the salmon module.

An association analysis was conducted between the mapping traits in **Table 4** and gene modules (**Figure 5C**). The results revealed that both the pink and purple modules exhibited a highly significant negative correlation with monoterpene M1 (p<0.01), while the black module showed a significant negative correlation with monoterpene M5 (p<0.05). The green and yellow modules demonstrated highly significant correlations (generally |r|>0.75 and p<0.001) with four straight-chain acetate esters (E1, E3, E4, E7), total ester content (TE), the five-year aroma sensory evaluation value (FS), and ethanol (AO1)—the precursor substrate for acetate ester synthesis. This suggests that these two modules may both harbor common candidate genes regulating the synthesis of multiple aroma compounds. Gene co-expression networks were constructed for each module (**Supplementary Figure S5**; **Supplementary Table S4**), identifying genes with high connectivity such as AGL103 (*Dil.03g026050.1*) and bZip1 (*Dil.01g036450.1*) in the green module (**Figure 5D**) and bHLH122 (*Dil.13g013320.1*) in the yellow module.

### 3.6 Candidate gene screening and expression pattern validation

**Figure 6:**
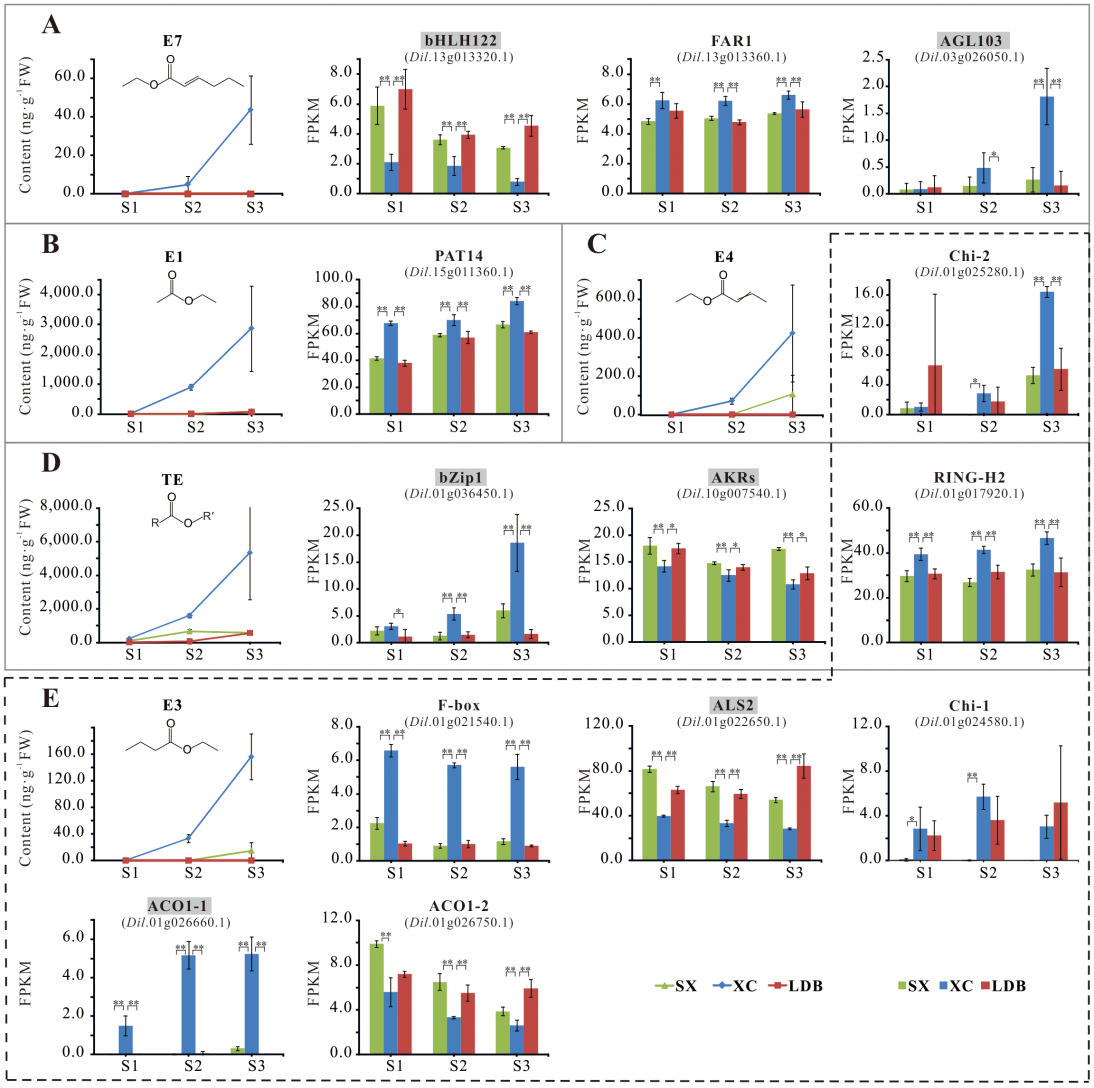
Accumulation of representative aroma compounds and differential expression of 13 candidate genes. **(A-E)** represents the content of E7, E1, E4, TE, E3, and the expression of candidate genes related to their synthesis, respectively. SX, XC, and LDB represent Shixia, Xiangcui, and Lidongben, respectively, while S1-S3 represent different stages of fruit development. FPKM values are calculated based on RNA-seq data from three biological replicates at each developmental stage. The names of key candidate genes are filled in gray. The * and ** represent significant (*P* < 0.05) and extremely significant (*P* < 0.01) differences, respectively.

Further analysis of correlations between gene expression and characteristic aroma compound contents across 17 developmental stages of 3 longan cultivars, combined with functional relevance assessment, revealed 13 candidate genes significantly or highly significantly associated with aroma synthesis (**Table 5**; **Figure 6**; **Supplementary Tables S2, S3**). These included an S-acyltransferase gene (*Dil*.15g011360.1) associated with ethyl acetate (E1); six genes linked to ethyl butyrate (E3): RING-H2 zinc finger protein (*Dil*.01g017920.1), acetolactate synthase (*Dil*.01g022650.1), endochitinases (*Dil*.01g024580.1, *Dil*.01g025280.1), and ACC oxidases (*Dil*.01g026660.1, *Dil*.01g026750.1), with endochitinase *Dil*.01g025280.1 also showing a significant positive correlation with ethyl crotonate (E4); three transcription factors associated with ethyl (E)-2-hexenoate (E7): bHLH122 (*Dil*.13g013320.1), FAR1 (*Dil*.13g013360.1), and AGL103 (*Dil*.03g026050.1); and three genes related to total ester content (TE): RING-H2 zinc finger protein (*Dil*.01g017920.1), bZip1 (*Dil*.01g036450.1), and aldo-keto reductase AKRs (*Dil*.10g007540.1).

**Table 5:** 13 candidate gene information related to the synthesis of longan aroma compounds.

Trait	QTL	Gene ID	Gene function	Related coefficient	Module
E1	E1-lm1917	*Dil*.15g011360.1	Probable protein S-acyltransferase 14 (*PAT14*)	0.644^**^	/
E3	E3-hk0913	*Dil*.01g021540.1	F-box/kelch-repeat protein At3g06240 (F-box)	0.501^*^	pink
	E3-lm2174	*Dil*.01g017920.1^a^	RING-H2 finger protein ATL56 (*RING-H2*)	0.814^**^	/
	E3-hk0614	*Dil*.01g022650.1^b^	Acetolactate synthase small subunit 2 (*ALS2*)	-0.616^**^	/
	E3-lm0748	*Dil*.01g024580.1^c^	Endochitinase EP3 (*Chi-1*)	0.583^*^	greenyellow
	E3-hk0613	*Dil*.01g025280.1^a,c^	Endochitinase EP3 (*Chi-2*)	0.824^**^	/
	E3-lm0888	*Dil.*01g026660.1^b,d^	1-aminocyclopropane-1-carboxylate oxidase homolog 1 (*ACO1-1)*	0.699^**^	green
	E3-lm0888	*Dil*.01g026750.1	1-aminocyclopropane -1-carboxylate oxidase homolog 1 (*ACO1-2)*	-0.681^**^	purple
E4	E4-hk0619	*Dil*.01g025280.1^a^	Endochitinase EP3 (*Chi-2*)	0.681^**^	/
E7	E7-np0842	*Dil*.13g013320.1^b^	Transcription factor bHLH122 (bHLH122)	-0.558^*^	yellow
	E7-np0842	*Dil*.13g013360.1	Protein FAR1-RELATED SEQUENCE 4 (FAR1)	0.611^**^	/
	E7-lm2298	*Dil*.03g026050.1^b^	Agamous-like MADS-box protein AGL103 (AGL103)	0.642^**^	green
TE	TE-lm2174	*Dil*.01g017920.1	RING-H2 finger protein ATL56 (RING-H2)	0.824^**^	/
	TE-lm0148	*Dil*.01g036450.1^b^	Basic leucine zipper 1 (bZip1)	0.791^**^	green
	TE-hk1131	*Dil.*10g007540.1^b^	Aldo-keto reductase family 4 member C10 (*AKRs*)	-0.609^*^	/

E1, E3, E4, E7 represent Ethyl Acetate, Ethyl butyrate, Ethyl crotonate and Ethyl 2-hexenoate; ^a^Genes were repeatedly located between different traits. ^b^Key candidate genes. ^c^Amino sugar and nucleotide sugar metabolism pathway. ^d^Butanoate metabolism pathway. The * and ** indicate that the correlation between gene expression and compound content reached significant (*P* < 0.05) and extremely significant (*P* < 0.01) levels in all 17 developmental stages of the three longan varieties, respectively.

During the critical S1-S3 stages of aroma formation in longan fruit (aroma profile begins to emerge at S2 stage in the strong-aroma cultivar, and the interval between adjacent stages was 10d), differential accumulation of characteristic aroma compounds (E1, E3, E4, E7) occurred alongside corresponding expression changes in candidate genes (**Figure 6**). At stage S3, endochitinase Chi-2 (*Dil*.01g025280.1) expression in the strong-aroma cultivar ‘Xiangcui’ (XC) was approximately triple that of non-aromatic cultivars SX and LDB, and E1 content in XC was 35-40 times higher. Expression of the ALS2 (*Dil*.01g022650.1) and bHLH122 (*Dil*.13g013320.1) decreased progressively during fruit development, with their expression in XC was approximately half and one-third of that in SX and LDB at S3, respectively. Concurrently, ethyl butyrate (E3) and ethyl (E)-2-hexenoate (E7) levels were significantly higher in XC.

Further combined with LOD value, Expl. % value, locus overlap stability, and direct correlation degree of gene function, the above 13 candidates genes related to the five aroma compound was re-evaluated. Six genes were ultimately identified as key candidate genes, including AKRs, ALS2, ACO1-1, transcription factors bHLH122, AGL103, and bZip1. These exhibited LOD scores of 3.80–5.52 and Expl.% values of 31.1%–41.8%.

## 4 Discussion

### 4.1 Obtaining and evaluating high-quality genetic maps

High-quality genetic linkage maps are fundamental for precise QTL mapping, and its evaluation indicators mainly include marker density, saturation, and ordering accuracy. Traditional genetic maps constructed using first- and second-generation markers (RFLP, AFLP, RAPD, SSR) typically contain only several hundred markers. These are labor-intensive to develop and suffer from low marker density, large intervals, and limited chromosomal position matching, resulting in poor candidate gene localization.

High-throughput SNP detection methods based on reduced-representation genome sequencing, whole-genome resequencing, or SNP arrays have become mainstream for building high-density genetic maps. Such SNP maps contain thousands or tens of thousands of markers, significantly improving density and saturation (Li P. et al., 2023). However, intervals ≥5 cM or 10 cM may persist, and generally ≤1 cM is considered suitable for gene mapping. Strategies to reduce intervals include combining second- and third-generation markers, expanding population size to increase recombination events, and constructing secondary populations for fine-mapping. Of course, if the marker intervals reside in genomic regions with identical parental backgrounds (preventing segregation in progeny) or assembly gaps, alternative approaches are required. Additionally, while marker order should theoretically align with physical positions, several factors can disrupt collinearity: different methods for dividing the marker into linkage group (according to LOD thresholds of linkage relationship or chromosomal origins), insufficient SNPs/Bin markers within contigs for orientation determination, chromosomal inversions, and regional variation in recombination rates (Chen Y. Y. et al., 2022).

This study constructed the first high-density Bin genetic map of longan using whole-genome resequencing of a ‘Shixia × Xiangcui’ population. The map comprises 15 linkage groups anchored by 3 517 Bin markers (representing 264 385 SNPs), spanning 1 666.79 cM at an average marker interval of 0.48 cM, demonstrating high density and uniform distribution. The numbers of mapped markers in this study far exceeded those in previous studies. For example, the framework map developed by Guo et al. (Guo et al., 2011) contained only 243 and 184 markers for the paternal and maternal parents, respectively, while exhibiting inconsistent linkage group numbers (19 and 20) relative to the haploid chromosome number (n=15). Although the map constructed by Jue et al. (Jue et al., 2021) using RAD-seq featured linkage groups matching the haploid chromosome count and with an average marker interval of 0.36 cM, it contained only 8 014 SNPs. The limited occurrence of gaps >5 cM (0.82%) in our map may stem from insufficient population size or localization within non-recombinant regions of the genome (Chen Y. Y. et al., 2022). Collinearity analysis revealed an average Spearman’s ρ of 0.870 between genetic and physical maps (**Table 2**), with ρ > 0.9 for LG1, 3, 6, 9-12 confirming consistent marker order. However, LG5 showed lower collinearity (ρ = 0.578), potentially due to its fewer SNPs (7 666 markers) reducing recombination events—which may compromise ordering accuracy. Alternative explanations include misassembled contigs or chromosomal translocations/inversions arising from divergent genetic backgrounds of the parental cultivars (Huang et al., 2020). Future efforts could employ F_2_ populations to increase recombination frequency and further improve map resolution.

### 4.2 Genome-wide distribution of QTLs for longan fruit aroma

This study represents the first quantitative trait locus (QTL) analysis of fruit aroma compounds in longan. Leveraging the high-density Bin map, 56 QTLs related to 9 compounds such as (E)-2-hexenal (A6), ethyl acetate (E1), ethanol (AO1) were identified in the whole genome of longan (**Table 4**). These QTLs exhibited LOD scores of 3.21–7.29 and explained phenotypic variation of 19.8%–51.0%. These QTLs were unevenly distributed across 11 linkage groups. Notably, two enriched regions—LG7: hk0743-lm0696 and LG8: hk0851-hk0585—harbored 36 QTLs, which were QTL hotspots controlling the synthesis of ethyl butanoate (E3), ethyl crotonate (E4), and ethyl (E)-2-hexenoate (E7). This phenomenon of QTLs clustering for structurally similarity or same biosynthetic pathway of volatile compounds has been reported in other fruit trees. For instance, Koyama et al. (Koyama et al., 2022) detected a QTL-rich region associated with 14 monoterpene contents in grape, which was located at the top of LG5 on the paternal and consensus maps and the QTLs were closely overlapped. Yang et al. (2023) detected 87 QTLs related to 15 volatiles on 14 linkage groups of apple. Among them, seven QTLs associated with four ester compounds were identified and confirmed on the same region of LG6.

The fruit aroma profile is complex and susceptible to maturity, light, temperature, soil and other factors (Sánchez et al., 2014); thus, multi-year assessment is essential. We conducted longitudinal evaluations of aroma traits in the ‘Shixia × Xiangcui’ population, collecting sensory evaluation data from 83 individuals across five years (2012–2015, 2018) and volatile compound detection from 48 individuals in 2018. These datasets were integrated to cross-validate QTL positions for enhanced accuracy. From the results, six of the eight QTLs for the five-year integrated sensory traits (FSP/FS) fell in two enrichment regions of the characteristic aroma compound traits. In addition, seven pleiotropic QTLs were identified, which did not include some partially overlapping QTLs. This co-localization suggests shared genetic regulation of closely related aroma compounds, such as ethyl butyrate (E3), ethyl crotonate (E4), and total esters content (TE). Collectively, these findings demonstrate robust reliability and stability of the mapped QTLs.

### 4.3 Key genes regulating ester aroma biosynthesis in longan

Ketoreductase (KRED), a member of the oxidoreductase family, catalyzes the reversible conversion between aldehydes/ketones and alcohols using coenzyme NAD(H) or NADP(H). KREDs is mainly divided into three groups: short-chain dehydrogenases/reductases (SDRs), medium-chain dehydrogenases/reductases (MDRs), and aldo-keto reductases (AKRs). While sharing similar catalytic functions, they exhibit structural and functional distinctions. Notably, the MDR family contains alcohol dehydrogenases (ADHs) (Persson et al., 2008). In this study, an AKRs gene (SwissProt-annotated) showing significant negative correlation with total ester content was identified at the LG9 TE-hk1131 locus, but it was annotated as an ADH enzyme (EC 1.1.1.2) that performs the same function in the KEGG database. We thus hypothesize it primarily catalyzes the conversion of ethanol (an important substrate for ethyl ester synthesis) to acetaldehyde in longan fruit: Ethanol + NADP^+^ ⇌ Acetaldehyde + NADPH + H^+^. Both AKRs and ADHs exhibit substrate specificity. For example, the enzyme kinetic analysis of grape *VvAdh2* and *VvAdh3* revealed differential binding affinities for ethanol versus acetaldehyde (Tesniere and Verries, 2000).

Acetolactate synthase (ALS, EC 2.2.1.6), also termed acetohydroxyacid synthase, comprises distinct large and small subunits (Liu et al., 2017). Here, the ALS small subunit ALS2 demonstrated a highly significant negative correlation with ethyl butyrate at the LG7 E3-hk0614 locus. Within plant butanoate metabolism (map00650), ALS catalyzes the first committed step in branched-chain amino acid synthesis, efficiently converting pyruvate to (S)-2-acetolactate. Alternatively, pyruvate undergoes oxidative decarboxylation to acetyl-CoA, which can be further converted to butanoyl-CoA—a precursor for ethyl butyrate synthesis via alcohol acyltransferase (AAT). We propose that ALS2 activity redirects pyruvate flux toward amino acid biosynthesis, thereby reducing ethyl butyrate accumulation.

ACC oxidase (ACO) and ACC synthase (ACS) are pivotal enzymes in ethylene biosynthesis. Ethylene-mediated aroma synthesis has received considerable attention. In ‘Fuji’ apple, ethylene release was positively correlated with total aroma content during cold storage, in which the ethyl butyrate and ethyl caproate were main volatile components (Qi et al., 2020). Defilippi et al. (Defilippi et al., 2004) reported that the esters biosynthesis in apple ACS/ACO gene-suppressed lines and fruits treated with 1-methylcyclopropene (1-MCP) was significantly reduced, and speculated that AAT enzyme gene was regulated by ethylene. Similarly, ethylene release could not be detected in the fruit of ACO-antisense apple plant, yet exogenous ethylene significantly promoted the production of volatile compounds such as esters and terpenes (Schaffer et al., 2007). Although non-climacteric fruits generate minimal ethylene, emerging evidence implicates ethylene in their ripening (Jiang et al., 2011). We identified ACO1-1 at LG7 E3-lm0888 locus, exhibiting strong positive correlation with ethyl butyrate and cultivar-specific expression in aromatic varieties. Notably, this locus harbors a tandem cluster of 16 ACO genes—mostly negatively correlated with ethyl butyrate—suggesting specialized roles in aroma development of non-climacteric longan. Furthermore, we discovered a tandem cluster of 16 ACO genes at this locus, most negatively correlated with ethyl butyrate content. Their specific roles in aroma formation in non-climacteric longan fruits are worthy of attention.

Transcriptional regulation of fruit ester biosynthesis, primarily through LOX pathway, involves MADS-box, bHLH, bZip, NAC and so on. Tomato MADS-box RIN directly regulates TomloxC, ADH2, and HPL, and rin mutants show reduced hexanal and (E)-2-hexenal during ripening (Qin et al., 2012). Apple *MdMADS24* activates *MdLOX1a*, enhancing ester accumulation in calli (Yue, 2020). Pear *PubHLH61*, *PuMYB91*, and *PuNAC100*-like synergistically positively regulate *PuLOX3* and *PuAAT*, thereby mediating the aroma of ‘Nanguo’ pear during cold storage (Luo, 2022). Banana *MabZip4/5* directly activates *BaAAT* promoters, with reduced expression diminishing aroma under refrigeration (Guo et al., 2018). In this study, bHLH122 and MADS-box AGL103 were identified at LG8:E7-np0842 and LG11:E7-lm2298 sites, which were significantly negatively correlated and positively correlated with ethyl (E)-2-hexenoate, respectively; And bZip1 at LG7:TE-lm0148 demonstrated significant positive correlation with the total content of esters. In our previous transcriptome analysis results, AGL103 and bZip1 were also identified as core transcription factors, which were significantly positively correlated 12 straight-chain esters including ethyl (E)-2-hexenoate (to be published separately).

Additional candidate genes were identified (**Table 5**), including S-acyltransferase PAT14 (protein palmitoylation) (Liu et al., 2024), RING-H2 zinc finger protein [ubiquitin-mediated degradation (Chen et al., 2022)], endochitinases Chi-1/-2 (β-1,4-glycosidic bond hydrolysis) (Gong et al., 2019), F-box protein (transcription factor specificity modulation) (Rieu et al., 2023), and FAR1 transcription factor (fruit development regulation) (Chen et al., 2021). These genes may indirectly influence aroma accumulation.

## 5 Conclusions

A first high-density Bin genetic map for longan was constructed using whole-genome resequencing, comprising 15 linkage groups with 3 517 Bin markers (264 385 SNPs). Spanning a total length of 1 666.79 cM, the map features an average marker interval of 0.48 cM, exceptional continuity (99.18% gaps <5 cM) and high collinearity (mean ρ=0.870). We mapped 56 QTLs associated with aroma trait and identified six key candidate genes regulating characteristic ester biosynthesis: aldo-keto reductase AKRs, acetolactate synthase small subunit ALS2, ACC oxidase ACO1-1, and transcription factors bHLH122, AGL103, and bZip1. These results are helpful to further understand the fruit aroma formation and provide valuable guidance for directed breeding of longan.

## Data Availability

The datasets presented in this study can be found in online repositories. The names of the repository/repositories and accession number(s) can be found in the article/**Supplementary Material**.

## References

[B1] Amarawathi Y. Singh R. Singh A. K. Singh V. P. (2008). Mapping of quantitative trait loci for basmati quality traits in rice (*Oryza sativa* L.). Mol. Breed. 21, 49–65. doi: 10.1007/s11032-007-9108-8

[B2] Bolger A. M. Lohse M. Usadel B. (2014). Trimmomatic: a flexible trimmer for Illumina sequence data. Bioinformatics 30, 2114–2120. doi: 10.1093/bioinformatics/btu170, PMID: 24695404 PMC4103590

[B3] Chen Y. Deng J. Chen J. Y. Zeng T. Yu T. T. Huang Q. H.. (2021). Genome-wide identification and expression analysis of FAR1/FHY3 transcription factor family in tomato. Plant Physiol. J. 57, 1983–1995. doi: 10.13592/j.cnki.ppj.2021.0061

[B4] Chen Y. Y. Schreiber M. Bayer M. M. Dawson I. K. Hedley P. E. Lei L.. (2022). The evolutionary patterns of barley pericentromeric chromosome regions. as shaped by linkage disequilibrium and domestication. Plant J. 111, 1580–1594. doi: 10.1111/tpj.15908, PMID: 35834607 PMC9546296

[B5] Chen S. J. Xu K. Kong D. Y. Wu L. Y. Chen Q. Ma X. S.. (2022). Ubiquitin ligase OsRINGzf1 regulates drought resistance by controlling the turnover of OsPIP2;1. Plant Biotechnol. J. 20, 1743–1755. doi: 10.1111/pbi.13857, PMID: 35587579 PMC9398399

[B6] Danecek P. Auton A. Abecasis G. Albers C. A. Banks E. DePristo M. A.. (2011). The variant call format and VCFtools. Bioinformatics 27, 2156–2158. doi: 10.1093/bioinformatics/btr330, PMID: 21653522 PMC3137218

[B7] Defilippi B. G. Kader A. A. Dandekar A. M. (2004). Apple aroma: alcohol acyltransferase.; a rate limiting step for ester biosynthesis, is regulated by ethylene. Plant Sci. 168, 1199–1210. doi: 10.1016/j.plantsci.2004.12.018

[B8] Di Pierro E. A. GianFranceschi L. Di Guardo M. Koehorst-van Putten H. J. Kruisselbrink J. W. Longhi S.. (2016). A high-density.; multi-parental SNP genetic map on apple validates a new mapping approach for outcrossing species. Horticult. Res. 3, 16057. doi: 10.1038/hortres.2016.57, PMID: 27917289 PMC5120355

[B9] Eduardo I. Chietera G. Pirona R. Pacheco I. Troggio M. Banchi E.. (2013). Genetic dissection of aroma volatile compounds from the essential oil of peach fruit: QTL analysis and identification of candidate genes using dense SNP maps. Tree Genet. Genomes 9, 189–204. doi: 10.1007/s11295-012-0546-z

[B10] Gong K. L. Chen S. H. Ji X. C. Lin Y. R. Zhang Q. (2019). The research progress of plant chitinases. Mol. Plant Breed. 17, 6840–6849. doi: 10.13271/j.mpb.017.006840

[B11] Guo Y. F. Zhang Y. L. Shan W. Cai Y. J. Liang S. M. Chen J. Y.. (2018). Identification of two transcriptional activators MabZIP4/5 in controlling aroma biosynthetic genes during banana ripening. J. Agric. Food Chem. 66, 6142–6150. doi: 10.1021/acs.jafc.8b01435, PMID: 29809003

[B12] Guo Y. S. Zhao Y. H. Fu J. X. Huang S. S. Wang Y. Lu B. B.. (2012). Identification of stable QTLs related to trunk girth in Longan. Sci. Horticult. 134, 248–252. doi: 10.1016/j.scienta.2011.11.007

[B13] Guo Y. S. Zhao Y. H. Liu C. M. (2011). QTLs analysis of several traits in Longan. Biotechnol. Biotechnol. Equip. 25, 2203–2209. doi: 10.5504/bbeq.2011.0014

[B14] Guo Y. S. Zhao Y. H. Liu C. J. Reng P. F. Huang T. L. Fu J. X.. (2009). Construction of a molecular genetic map for Longan based on RAPD, ISSR, SRAP and AFLP markers. Acta Hortic. Sin. 36, 655–662. doi: 10.16420/j.issn.0513-353x.2009.05.006

[B15] Hu W. S. Huang A. P. Jiang F. Jiang J. M. Chen X. P. Zheng S. Q. (2015). Identification and genetic diversity of reciprocal hybrids in longan (*Dimocarpus longan*) by SSR. Acta Hortic. Sin. 42, 1899–1908. doi: 10.16420/j.issn.0513-353x.2015-0131

[B16] Huang G. Wu Z. Percy R. G. Bai M. Z. Li Y. Frelichowski J. E.. (2020). Genome sequence of Gossypium herbaceum and genome updates of Gossypium arboreum and Gossypium hirsutum provide insights into cotton A-genome evolution. Nat. Genet. 52, 516–524. doi: 10.1038/s41588-020-0607-4, PMID: 32284579 PMC7203013

[B17] Jiang T. M. Yin X. R. Wang P. Sun C. D. Xu C. J. Li X.. (2011). Research advance in regulation of ethylene during ripening and senescence of non-climacteric fruit. Acta Hortic. Sin. 38, 371–378. doi: 10.16420/j.issn.0513-353x.2011.02.023

[B18] Jo H. Koh G. (2015). Faster single-end alignment generation utilizing multi-thread for BWA. Bio-med. Mater. Eng. 26, S1791–S1796. doi: 10.3233/BME-151480, PMID: 26405948

[B19] Jue D. W. Liu L. Q. Sang X. L. Shu B. Wang J. H. Wang Y. C.. (2021). SNP-based high-density genetic map construction and candidate gene identification for fruit quality traits of Dimocarpus longan Lour. Sci. Hortic. 284, 110086. doi: 10.1016/j.scienta.2021.110086

[B20] Kiszonas A. M. Boehm Jeffrey D. See D. Morris C. F. (2017). Identification of SNPs, QTLs and dominant markers associated with wheat grain flavor using genotyping-by-sequencing. J. Cereal Sci. 76, 140–147. doi: 10.1016/j.jcs.2017.06.006

[B21] Koyama K. Kono A. Ban Y. Bahena-Garrido S. M. Ohama T. Iwashita K.. (2022). Genetic architecture of berry aroma compounds in a QTL (quantitative trait loci) mapping population of interspecific hybrid grapes (*Vitis labruscana* × *Vitis vinifera*). BMC Plant Biol. 22, 458. doi: 10.1186/s12870-022-03842-z, PMID: 36151514 PMC9503205

[B22] Lander E. S. Botstein D. (1989). Mapping Mendelian factors underlying quantitative traits using RFLP markers. Genetics 121, 185–199. doi: 10.1093/genetics/121.1.185, PMID: 2563713 PMC1203601

[B23] Li F. M. Liu Z. Y. Chen H. X. Wu J. Cai X. Wang H.. (2023). QTL mapping of leaf-related traits using a high-density bin map in Brassica rapa. Horticulturae 9, 433. doi: 10.3390/horticulturae9040433

[B24] Li P. Liu R. T. Tan X. B. Zhang Y. Liu C. H. (2023). Research progress in genetic map construction and QTL mapping for disease resistance in grapevine. J. Fruit Sci. 40, 1245–1254. doi: 10.13925/j.cnki.gsxb.20220031

[B25] Lin Y. L. Min J. M. Lai R. L. Wu Z. Y. Chen Y. K. Yu L. L.. (2017). Genome-wide sequencing of longan (Dimocarpus longan Lour.) provides insights into molecular basis of its polyphenol-rich characteristics. Gigascience 5, 1–14. doi: 10.1093/gigascience/gix023, PMID: 28368449 PMC5467034

[B26] Liu Y. D. Li Y. Y. Wang X. Y. (2017). Molecular evolution of acetohydroxyacid synthase in bacteria. Microbiologyopen 6, 524. doi: 10.1002/mbo3.524, PMID: 28782269 PMC5727371

[B27] Liu F. Qu P. Y. Li J. P. Yang L. N. Geng Y. J. Lu J. Y.. (2024). Arabidopsis protein S-acyl transferases positively mediates BR signaling through S-acylation of BSK1. Proc. Natl. Acad. Sci. United States America 121, e2322375121. doi: 10.1073/pnas.2322375121, PMID: 38315835 PMC10873554

[B28] Luo M. L. (2022). Molecular mechanism of different transcription factors synergistically regulating key genes of ester biosynthesis to mediate aroma loss in cold-stored ‘Nanguo’ pears. Ph.D. Thesis (Shenyang, China: Shenyang Agricultural University). doi: 10.27327/d.cnki.gshnu.2022.000003

[B29] Ma X. Fan L. Zhang Z. F. Yang X. Liu Y. C. Ma Y. M.. (2023). Global dissection of the recombination landscape in soybean using a high-density 600K SoySNP array. Plant Biotechnol. J. 21, 606–620. doi: 10.1111/pbi.13975, PMID: 36458856 PMC9946146

[B30] Pereira L. Ruggieri V. Pérez S. Alexiou K. G. Fernández M. Jahrmann T.. (2018). QTL mapping of melon fruit quality traits using a high-density GBS-based genetic map. Plant Biol. 18, 324. doi: 10.1186/s12870-018-1537-5, PMID: 30509167 PMC6278158

[B31] Persson B. Hedlund J. Jornvall H. (2008). The MDR superfamily. Cell. Mol. Life Sci. 65, 3879–3894. doi: 10.1007/s00018-008-8587-z, PMID: 19011751 PMC2792335

[B32] Peter L. Steve H. (2008). WGCNA: an R package for weighted correlation network analysis. BMC Bioinf. 9, 1–32. doi: 10.1186/1471-2105-9-559, PMID: 19114008 PMC2631488

[B33] Qi W. Y. Wang H. J. Zhou Z. Yang P. Wu W. B. Li Z. M.. (2020). Ethylene emission as a potential indicator of Fuji apple flavor quality evaluation under low temperature. Hortic. Plant J. 6, 231–239. doi: 10.1016/j.hpj.2020.03.007

[B34] Qin M. F. Li L. T. Singh J. Sun M. Y. Bai B. Li S. W.. (2022). Construction of a high-density bin-map and identification of fruit quality-related quantitative trait loci and functional genes in pear. Horticult. Res. 9, uhac141. doi: 10.1093/hr/uhac141, PMID: 36072841 PMC9437719

[B35] Qin G. Z. Wang Y. Y. Cao B. H. Wang W. H. Tian S. P. (2012). Unraveling the regulatory network of the MADS box transcription factor RIN in fruit ripening. Plant J. 70, 243–255. doi: 10.1111/j.1365-313X.2011.04861.x, PMID: 22098335

[B36] Rey-Serra P. Mnejja M. Monfort A. (2022). Inheritance of esters and other volatile compounds responsible for the fruity aroma in strawberry. Front. Plant Sci. 13. doi: 10.3389/fpls.2022.959155, PMID: 36035685 PMC9412188

[B37] Rieu P. Turchi L. Thévenon E. Zarkadas E. Nanao M. Chahtane H.. (2023). The F-box protein UFO controls flower development by redirecting the master transcription factor LEAFY to new cis-elements. Nat. Plants 9, 315–329. doi: 10.1038/s41477-022-01336-2, PMID: 36732360

[B38] Sánchez G. Martínez J. Romeu J. García J. Monforte A. J. Badenes M. L.. (2014). The peach volatilome modularity is reflected at the genetic and environmental response levels in a QTL mapping population. BMC Plant Biol. 14, 137. doi: 10.1186/1471-2229-14-137, PMID: 24885290 PMC4067740

[B39] Schaffer R. J. Friel E. N. Souleyre E. J. F. Bolitho K. Thodey K. Ledger S.. (2007). A genomics approach reveals that aroma production in apple is controlled by ethylene predominantly at the final step in each biosynthetic pathway. Plant Physiol. 144, 1899–1912. doi: 10.1104/pp.106.093765, PMID: 17556515 PMC1949883

[B40] Shannon P. Markiel A. Ozier O. Baliga N. S. Wang J. T. Ramage D.. (2003). Cytoscape: a software environment for integrated models of biomolecular interaction networks. Genome Res. 13, 2498–2504. doi: 10.1101/gr.1239303, PMID: 14597658 PMC403769

[B41] Souleyre E. J. F. Nieuwenhuize N. J. Wang M. Y. Winz R. A. Matich A. J. Ileperuma N. R.. (2022). Alcohol acyl transferase genes at a high-flavor intensity locus contribute to ester biosynthesis in kiwifruit. Plant Physiol. 190, 1100–1116. doi: 10.1093/plphys/kiac316, PMID: 35916752 PMC9516725

[B42] Sun Y. H. Li X. Z. Ma Z. Y. Chen S. X. (2022). Quantitative trait locus mapping of fruit aroma compounds in cucumber (*Cucumber sativus* L.) based on a recombinant inbred line population. Horticult. Res. 9, 2960–2973. doi: 10.1093/hr/uhac151, PMID: 36196068 PMC9527598

[B43] Tang H. B. Zhang X. T. Miao C. Y. Zhang J. S. Ming R. Schnable J. C.. (2015). ALLMAPS: robust scaffold ordering based on multiple maps. Genome Biol. 16, 3. doi: 10.1186/s13059-014-0573-1, PMID: 25583564 PMC4305236

[B44] Tesniere C. Verries C. (2000). Molecular cloning and expression of cDNAs encoding alcohol dehydrogenases from *vitis vnifera* L. during berry development. Plant Sci. 157, 77–88. doi: 10.1016/s0168-9452(00)00274-0, PMID: 10940471

[B45] Tikunov Y. Roohanitaziani R. Meijer-Dekens F. Molthoff J. Paulo J. Finkers R.. (2020). The genetic and functional analysis of flavor in commercial tomato: the FLORAL4 gene underlies a QTL for floral aroma volatiles in tomato fruit. Plant J. 103, 1185–1204. doi: 10.1111/tpj.14795, PMID: 32369642 PMC7496274

[B46] Van Ooijen J. W. (2009). MapQTL 6.0, software for the mapping of quantitative trait loci in experimental populations of diploid species [Z] (Wageningen, Netherlands: Kyazma B.V).

[B47] Voorrips R. E. (2002). MapChart: software for the graphical presentation of linkage maps and QTLs. J. Heredity 93, 77–78. doi: 10.1093/jhered/93.1.77, PMID: 12011185

[B48] Yang S. B. Yu J. Yang H. J. Zhao Z. Y. (2023). Genetic analysis and QTL mapping of aroma volatile compounds in the apple progeny ‘Fuji’×’Cripps Pink’. Front. Plant Sci. 14. doi: 10.3389/fpls.2023.1048846, PMID: 37021304 PMC10067597

[B49] Yu Y. Bai J. H. Chen C. X. Plotto A. Yu Q. B. Baldwin E. A.. (2017). Identification of QTLs controlling aroma volatiles using a ‘Fortune’×’Murcott’ (*Citrus reticulata*) population. BMC Genomics 18, 646. doi: 10.1186/s12864-017-4043-5, PMID: 28830354 PMC5568196

[B50] Yu G. C. Wang L. G. Han Y. Y. Yu Q. (2012). clusterProfiler: an R package for comparing biological themes among gene clusters. Omics 16, 284–287. doi: 10.1089/omi.2011.0118, PMID: 22455463 PMC3339379

[B51] Yue S. S. (2020). Study on apple ethylene response factor *ERF1B* regulating aroma biosynthesis in LOX pathway. Ph.D. Thesis (Taian, China: Shandong Agricultural University). doi: 10.27277/d.cnki.gsdnu.2020.000588

[B52] Zeng Y. Wang M. Y. Hunter D. C. Matich A. J. McAtee P. A. Knäbel M.. (2020). Sensory-directed genetic and biochemical characterization of flavor-related volatile terpene production in ripe kiwifruit. Plant Physiol. 183, 51–66. doi: 10.1104/pp.20.00186, PMID: 32184346 PMC7210626

[B53] Zhao L. Zhang Y. Wei X. D. Liang W. H. Zhao C. F. Zhou L. H.. (2022). Mapping of QTLs for chlorophyll content in flag leaves of rice on high-density bin map. Sci. Agricult. Sin. 55, 825–836. doi: 10.3864/j.issn.0578-1752.2022.05.001

[B54] Zheng S. Q. Zeng L. H. Zhang J. S. Lin H. T. Deng C. J. Zhuang Y. M. (2019). Fruit scientific research in New China in the past 70 years: Longan. J. Fruit Sci. 36, 1414–1420. doi: 10.13925/j.cnki.gsxb.Z15

[B55] Zhou Z. Q. Zhang C. S. Zhou Y. Hao Z. F. Wang Z. H. Zeng X.. (2016). Genetic dissection of maize plant architecture with an ultra-high density bin map based on recombinant inbred lines. Genomics 17, 178. doi: 10.1186/s12864-016-2555-z, PMID: 26940065 PMC4778306

