# Supplementary material for: Construction of high-density bin genetic map and QTL mapping of fruit aroma in longan (Dimocarpus longan Lour.)
Source: Front Plant Sci. 2025 Aug 14;16:1642854. doi: 10.3389/fpls.2025.1642854 (PMC12391000; doi:10.3389/fpls.2025.1642854)
Supplement: Supplementary file 1 [file DataSheet1.doc]

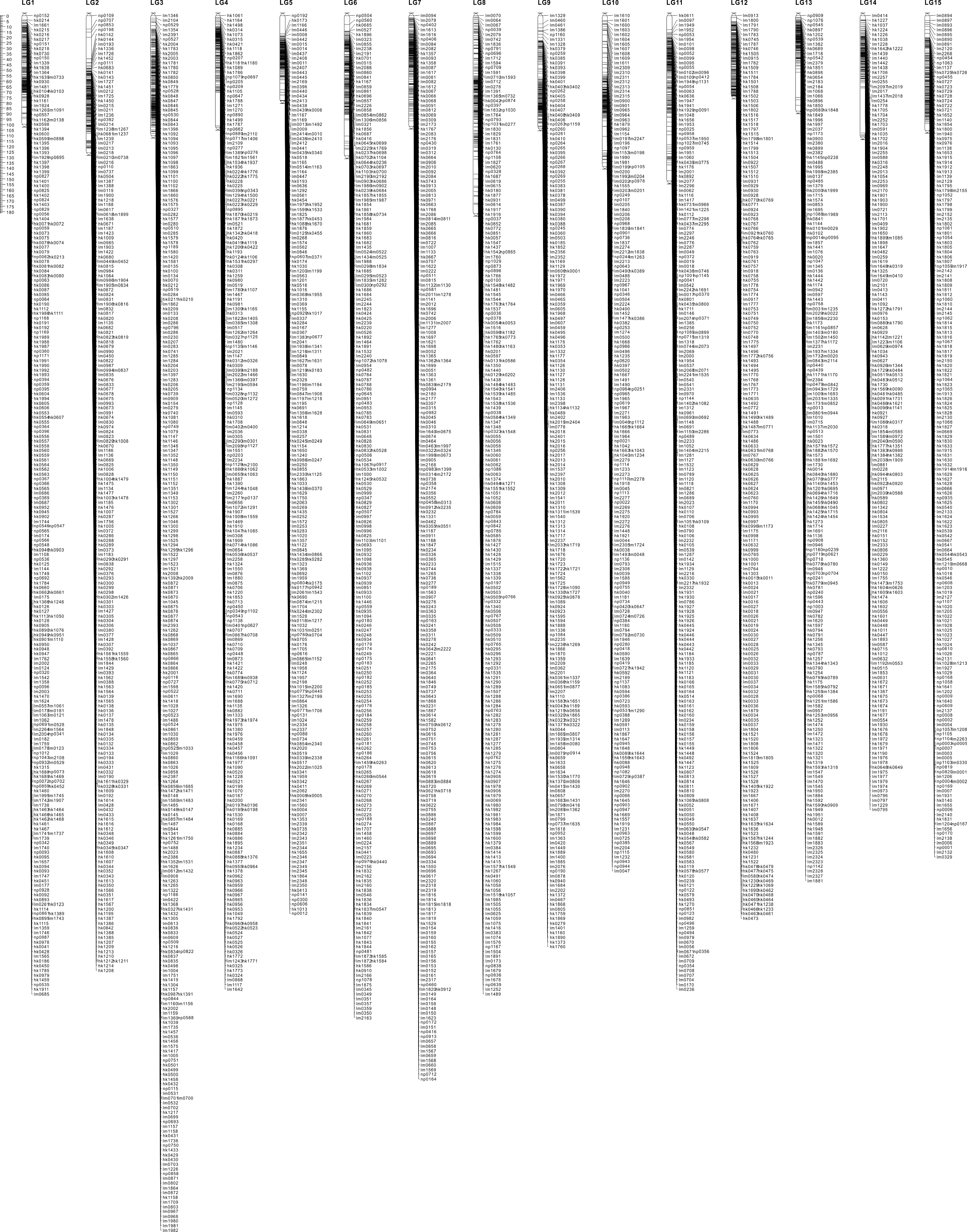


**Supplementary Figure S1.** High-density genetic linkage map of longan (3 517 Bin markers)


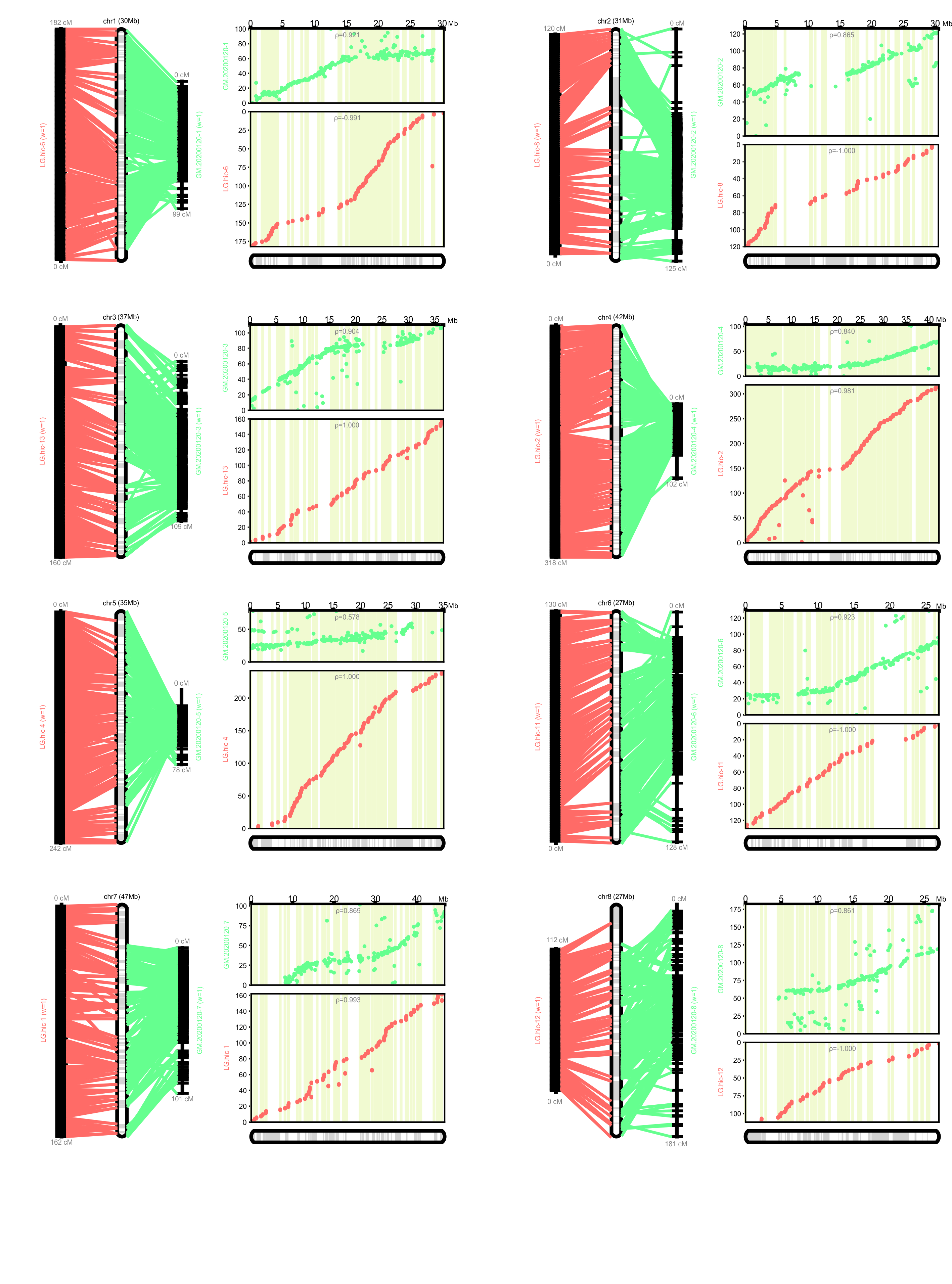


**Supplementary Figure S2.** Collinearity visualization between genetic map and HiC-anchored genome map (Chr1-8)


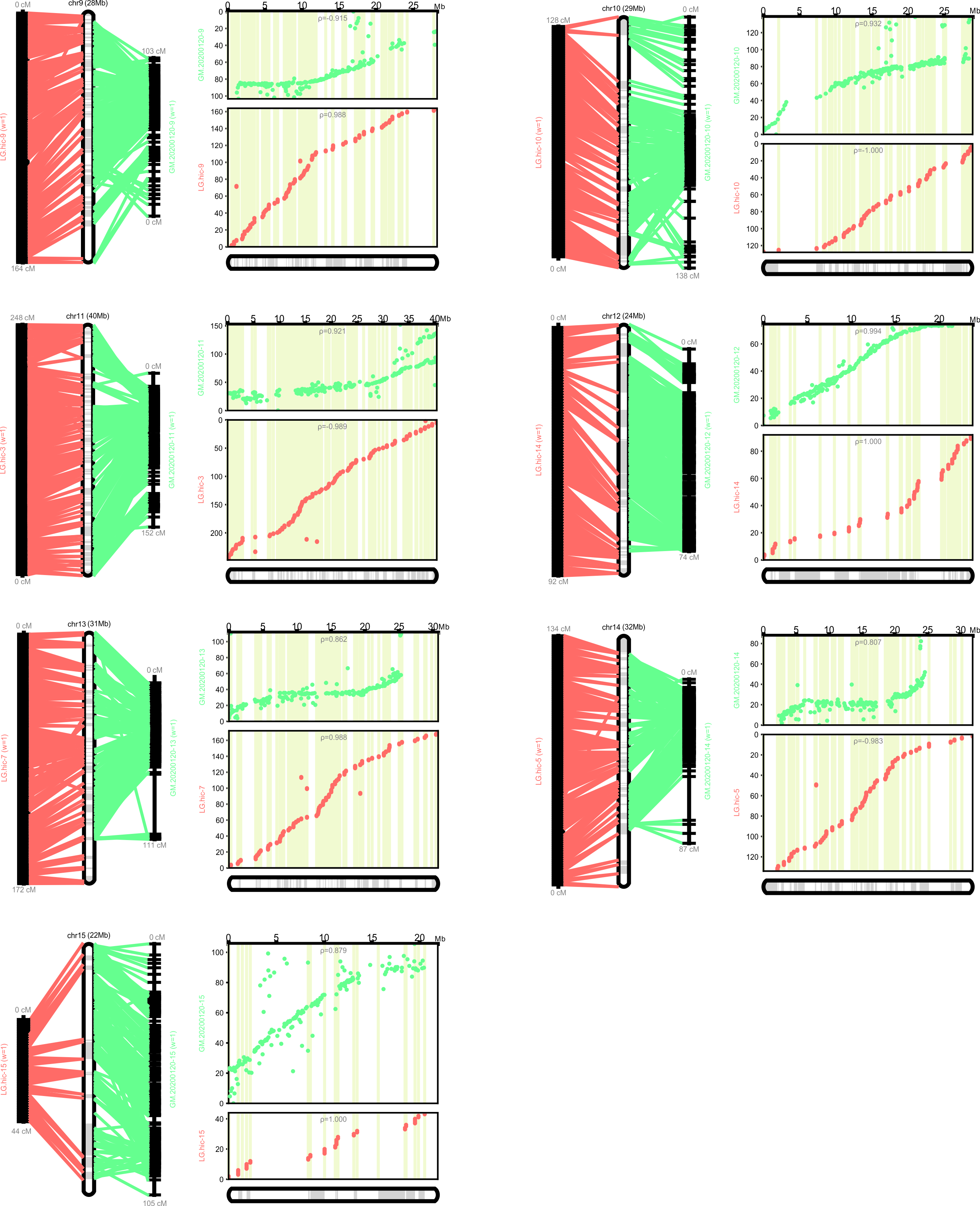


**Supplementary Figure S3.** Collinearity visualization between genetic map and HiC-anchored genome map (Chr9-15)


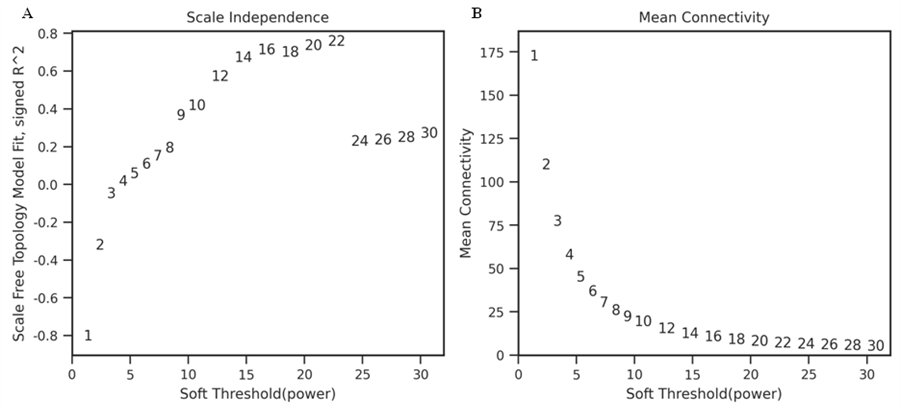


**Supplementary Figure S4.** Determination of soft-thresholding power β. A, scale-free topology fit index as a function of the soft-thresholding power. B, the ordinate represents the mean connectivity of each soft threshold.


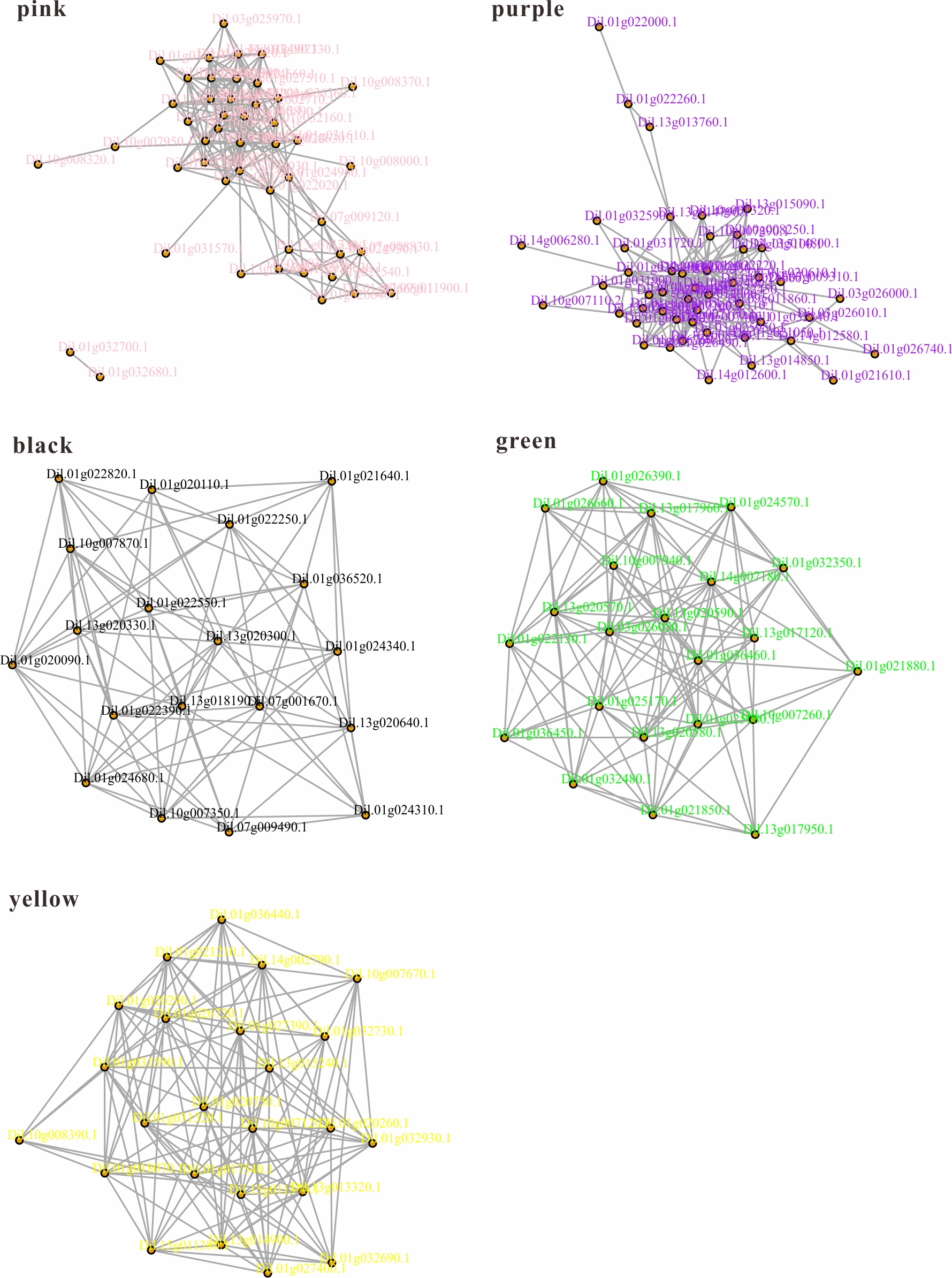


**Supplementary Figure S5.** Gene co-expression networks within pink, purple, black, green and yellow modules.
